# Assembly of a Metal–Organic Framework (MOF) Membrane on a Solid Electrocatalyst: Introducing Molecular‐Level Control Over Heterogeneous CO_2_ Reduction

**DOI:** 10.1002/anie.202102320

**Published:** 2021-05-05

**Authors:** Subhabrata Mukhopadhyay, Ran Shimoni, Itamar Liberman, Raya Ifraemov, Illya Rozenberg, Idan Hod

**Affiliations:** ^1^ Department of Chemistry and Ilse Katz Institute for Nanoscale Science and Technology Ben-Gurion University of the Negev Beer-Sheva 8410501 Israel

**Keywords:** CO_2_ reduction, electrocatalyst, mass Transport, metal–organic framework (MOF), UiO-66

## Abstract

Electrochemically active Metal‐Organic Frameworks (MOFs) have been progressively recognized for their use in solar fuel production schemes. Typically, they are utilized as platforms for heterogeneous tethering of exceptionally large concentration of molecular electrocatalysts onto electrodes. Yet so far, the potential influence of their extraordinary chemical modularity on electrocatalysis has been overlooked. Herein, we demonstrate that, when assembled on a solid Ag CO_2_ reduction electrocatalyst, a non‐catalytic UiO‐66 MOF acts as a porous membrane that systematically tunes the active site's immediate chemical environment, leading to a drastic enhancement of electrocatalytic activity and selectivity. Electrochemical analysis shows that the MOF membrane improves catalytic performance through physical and electrostatic regulation of reactants delivery towards the catalytic sites. The MOF also stabilizes catalytic intermediates via modulation of active site's secondary coordination sphere. This concept can be expanded to a wide range of proton‐coupled electrochemical reactions, providing new means for precise, molecular‐level manipulation of heterogeneous solar fuels systems.

## Graphical abstract

Metal–Organic Frameworks (MOFs) assembled on a solid electrocatalyst can act as porous membrane to tune the active site's immediate chemical environment, leading to improved electrocatalytic performance and selectivity. A UiO‐66 MOF‐membrane enhances CO_2_‐to‐CO catalysis when grown over Ag. It stabilizes reactive intermediates and attenuates reactant's mass transport.

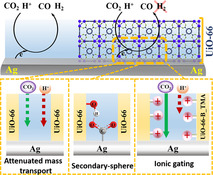

## Introduction

Metal‐Organic Frameworks (MOFs) have attracted significant attention from the scientific community over the last two decades.^[1]^ Due to their enormous surface area, high porosity, and the scope of reticular control over their chemical, electronic and photophysical properties, MOFs are often used in various applications, such as gas sorption,^[2]^ gas separation,^[3]^ catalysis,^[4]^ sensing,^[5]^ and artificial photosynthesis.^[6]^

In recent years, MOFs have also shown great potential to be utilized in electrocatalytic energy‐related schemes.^[7]^ Generally, electrocatalytic MOFs possess several key advantages over conventional, dense heterogeneous solids.[^6d^, ^8^] They can potentially be used as a porous platform for the assembly of extremely high density of catalytically active sites (molecular catalysts) while providing mass‐transport conduits accessible for diffusion of ions and catalytic‐substrates towards the active sites.^[9]^ Consequently, research efforts have mainly focused on implementing electrochemically active MOFs as the catalytic component in the electrocatalytic cell, either through (a) the use of the MOF structural elements themselves (ligands or nodes) as electroactive catalysts,^[10]^ or (b) the immobilization of active molecular catalysts within the MOF pores.[^9c^, ^11^]

Yet, one can also consider the well‐defined structure and chemical modularity of MOFs as another important virtue for efficient electrocatalysis, as it can be used to fine‐tune the immediate chemical environment of the active site, and thus affect its overall catalytic performance.^[12]^ In fact, in biological systems, catalytic enzymes have evolved chemical and structural mechanisms that modulate the surroundings of the active site and hence regulate the activity and selectivity of a desired catalytic reaction. For instance, membrane proteins have provided precise control over the rate of proton delivery towards the active site in order to regulate redox‐based proton‐coupled electron transfer (PCET) reactions.^[13]^ Moreover, an enzyme may modulate the secondary coordination sphere of the active site via the incorporation of pendant functional groups, such as proton relays or charge‐bearing moieties.^[14]^ These groups play a crucial role during catalysis, as they may stabilize the intermediate species and enhance the reaction rate and selectivity.

Inspired by these concepts, we realized that another approach could be implemented, where a non‐electrocatalytic MOF would be used as a porous membrane layered over a solid heterogeneous electrocatalyst. Following this principle, a suitably designed MOF membrane has the potential to modify the microenvironment of the underlying heterogeneous catalyst and affect its electrocatalytic properties in a wide variety of PCET reactions.

In this work, as a model PCET Scheme, we chose to focus on the electrochemical reduction of CO_2_ ^[15]^ using an Ag electrocatalyst. Ag is well known for its ability to effectively catalyze the reduction of CO_2_ into CO.^[16]^ Nevertheless, proton reduction to H_2_ constitutes a competitive side‐reaction, often impeding the activity and selectivity of the desired conversion of CO_2_.^[17]^ Consequently, a favorable activation of CO_2_ over H^+^ is a requisite for an efficient Ag‐based electrocatalytic system. To this end, we prepared a thin layer of Zr_6_‐oxo based MOF (UiO‐66), directly assembled on an Ag CO_2_ reduction electrocatalyst. We found that the electrochemical CO_2_ reduction properties of the Ag catalyst were precisely tuned by the MOF overlayer, leading to a drastic improvement in electrocatalytic activity and selectivity. Detailed electrochemical investigation and material characterizations reveal that the effect of the MOF layer is threefold. First, it serves as a porous membrane that physically attenuates the mass transport of reactants (CO_2_, H^+^) towards the Ag electrocatalyst. By doing so, the local concentrations of reactants at the vicinity of the catalytic sites are significantly altered compared to those in the bulk solution, thus shifting catalysis pathways. Second, the MOF's Zr_6_‐oxo nodes contain pendant Brønsted acidic groups proximal to the catalytically active surface; these groups accelerate electrocatalysis through the stabilization of activated *COO^−^ intermediates. Third, post‐synthetic modification of the MOF with a positively charged ligand, (3‐carboxypropyl)trimethylammonium (TMA), imposes an electrostatic ionic gating mechanism yielding finer control over the H^+^ flux towards the catalyst surface. Overall, the combination of all 3 mechanisms allowed the systematic tuning of CO_2_‐to‐CO selectivity from 43 % for bare Ag, up to 89 % (at a potential of −0.8 V vs. RHE).

## Results and Discussion

**Figure 1:**
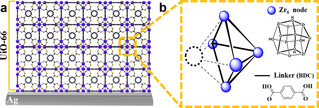
a) Illustration of the structure of a UiO‐66 thin film assembled on an Ag electrocatalyst. b) Illustration of a missing‐cluster (MC) defect in UiO‐66.

Thin films of UiO‐66 were grown on flat Ag electrocatalysts, as illustrated in Figure 1 a. The synthetic strategy was adapted from a previously reported synthetic procedure (for experimental details, see Supporting Information).^[18]^ In short, a DMF solution of ZrOCl_2_, acetic acid, and 1,4‐benzene‐di‐carboxylic acid (BDC) was drop‐casted on a flat Ag catalyst. Thereafter, the Ag foil was placed in a closed container and kept at 110 °C in the presence of DMF and acetic acid vapor for 4 hours.

**Figure 2:**
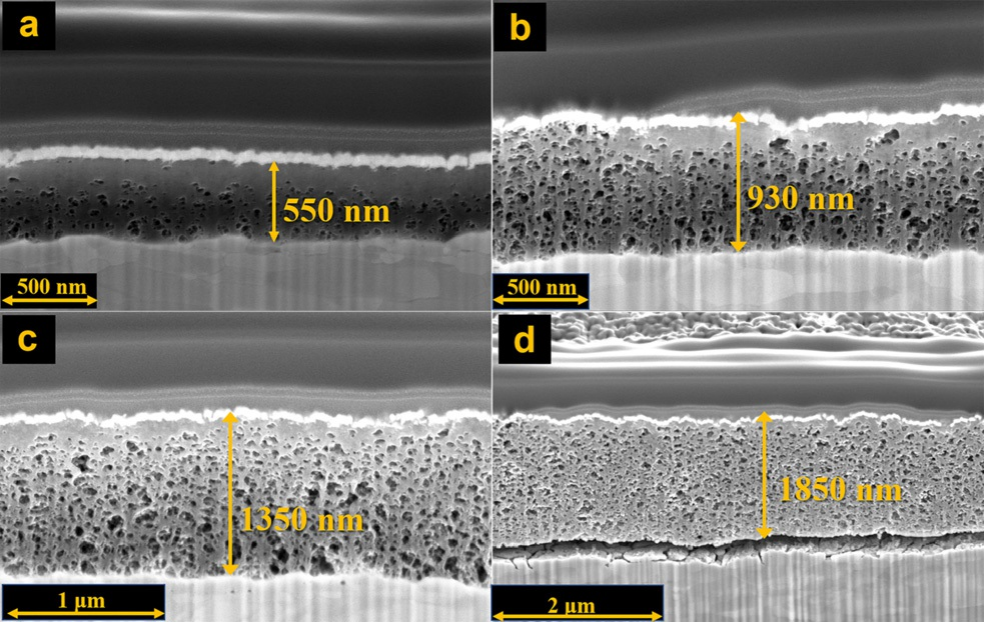
SEM‐FIB cross‐section images of a) UiO‐66‐A, b) UiO‐66‐B, c) UiO‐66‐C and d) UiO‐66‐D, coated on flat Ag electrodes.

Scanning electron microscopy (SEM) images show that the UiO‐66 grown on Ag formed continuous, homogeneous, and porous, thin films (Figure S1). By increasing the volume of precursor solution drop‐casted on the Ag film, we were able to obtain four samples with increasing UiO‐66 thickness (Figure S2, and Table S1). Using SEM‐FIB (FIB=Focused Ion Beam) analysis (Figure 2, and Figure S2), we determined the film thicknesses to be 550 nm, 930 nm, 1350 nm, and 1850 nm for samples UiO‐66‐A, UiO‐66‐B, UiO‐66‐C and UiO‐66‐D, respectively.

**Figure 3:**
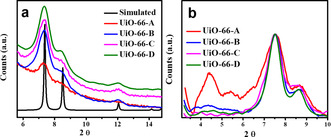
PXRD patterns of UiO‐66‐(A‐D) (a) and the simulated PXRD pattern of UiO‐66 and b) UiO‐66‐(A‐D) recorded in the 2*θ* region of 3–10°.

For all samples, powder X‐ray diffraction (PXRD) confirms the successful growth of UiO‐66 thin films (Figure 3 a). Moreover, a careful analysis at low angles reveals two additional broad peaks located at low 2*θ* (4.4° and 5.4°), indicating the presence of nanoregions of MC defects in all four UiO‐66 thin films (Figure 3 b).^[19]^ UiO‐66‐A exhibits the most intense signals in this region of the PXRD pattern, thus suggesting a higher density of MC defects in this sample. N_2_ physisorption (Figure S3) measurements reveal that the BET surface area of UiO‐66‐A is considerably lower compared to the surface area of UiO‐66‐(B‐D) (Table S2), as a result of the excessively high degree of defects in UiO‐66‐A.^[19d]^ The surface area of UiO‐66‐(B‐D) matches well with literature reports of defective UiO‐66.[^9^, ^19^]

**Figure 4:**
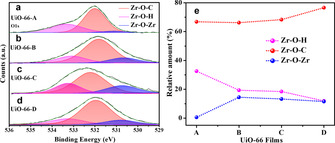
O1s XPS spectra of a) UiO‐66‐A, b) UiO‐66‐B, c) UiO‐66‐C and d) UiO‐66‐D, respectively; e) percentage of oxygen contained in “Zr‐OH”, “Zr‐O‐C”, and “Zr‐O‐Zr” in the (UiO‐66–Ag) interface for UiO‐66‐(A‐D), as determined from the O1s XPS spectra.

The O1s X‐ray photoelectron spectroscopy (XPS) further confirms the presence of MC defects in all samples (Figure 4, Figure S4 and S5). Typically, the O1s signal of UiO‐66 shows 3 distinct peaks, each assigned to a specific oxygen‐based species at the Zr_6_‐oxo node:^[20]^ “Zr‐O‐Zr” (530.5±0.2 eV), “Zr‐O‐H” (533.4±0.2 eV), and “Zr‐O‐C” (532.0±0.1 eV), i.e., Zr_6_‐oxo nodes, exposed hydroxy groups, and BDC linkers present within the UiO‐66 layers, respectively.

For all samples, the percentages of each oxygen‐based species are plotted in Figure 4 e. UiO‐66‐A clearly presents a lower relative density of “Zr‐O‐Zr” than the rest of the samples, which all exhibit a rather similar “Zr‐O‐Zr” relative density. The relative density of Zr_6_‐oxo nodes is inversely correlated to the relative density of MC defects; thus, one can monitor the extent of MC defects by probing the “Zr‐O‐Zr” signal. In other words, among all samples, UiO‐66‐A contains the largest number of MC defects, in agreement with the PXRD results. Furthermore, generally an MC defect is characterized by a missing Zr_6_‐oxo cluster alongside all of its 12 associated linkers,^[19b]^ thus leaving behind neighboring Zr_6_‐oxo clusters with high density of missing linkers and a corresponding large concentration of exposed pendant, Brønsted acidic OH groups, as illustrated in Figure 1 b. Indeed, the relative intensity of the “Zr‐O‐H” O1s peak increases with increasing density of MC defects (Figure 4 e), reaching a maximum for UiO‐66‐A. As seen in Figure S5, this notion was further supported via determination of Zr_6_ nodes density for UiO‐66‐(A‐D) via inductively coupled plasma optical emission spectrometry (ICP‐OES). It shows a considerably lower Zr_6_ node density for UiO‐66‐A while the density of Zr_6_ node in UiO‐66‐(B‐D) is comparable to each other. Thus, we were able to tune the thickness and chemical composition of UiO‐66 thin films grown on Ag electrodes, which in turn will serve to modulate their respective electrocatalytic CO_2_ reduction activity, as will be further discussed below.

We then set to examine the effect of the MOF coating on the electrocatalytic CO_2_ reduction properties of the underlying Ag catalyst. Electrochemical analysis was carried out using a gas‐tight H‐cell three‐electrode setup, in CO_2_‐saturated 0.1 M NaHCO_3_ electrolyte (pH 6.8). Platinum foil, Ag/AgCl (saturated KCl), and UiO‐66‐coated Ag foils were used as counter, reference, and working electrodes, respectively. The working and reference electrodes were separated from the counter electrode by an ion‐exchange membrane, Nafion 117 (see Supporting Information Experimental Section for details).

**Figure 5:**
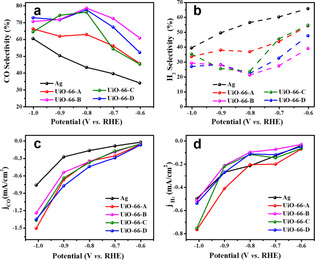
Variation in product selectivity of bare Ag and Ag coated with UiO‐66‐(A‐D) towards a) CO and b) H_2_ at different applied potentials. Variation of partial catalytic currents of c) CO_2_ reduction (*j*_CO_) and d) H_2_ evolution (*j* ${}_{H{}_{2}}$ ) at different applied potentials.

The electrocatalytic activity of bare Ag and all UiO‐66‐coated electrodes were measured through bulk electrolysis experiments within a potential window of −0.6 V to −1.0 V vs. RHE (Figure S6). As can be seen in Figure 5 a,b, a drastic improvement in electrocatalytic selectivity towards CO production was achieved for all UiO‐66‐modified electrodes compared to a bare Ag foil. Notably, at the potential of −0.8 V vs. RHE, the CO production selectivity was systematically enhanced from 43 % for bare Ag up to 79 % for UiO‐66‐B (we note that for all experiments, the combined (CO + H_2_) faradaic efficiency was essentially 100 %, as seen in Figure S7). It is important to mention that the selectivity towards CO production by bare Ag can vary widely depending upon several important parameters as the type of electrolyte solution,[^15e^, ^15f^] surface exposed crystal facets,^[15g]^ porosity of the catalytic surface,^[15h]^ crystallinity and particle sizes,[^15i^, ^15j^] and surface roughness.^[16b]^ Moreover, the partial catalytic current densities corresponding to CO (*j*_CO_) and H_2_ (*j* ${}_{H{}_{2}}$ ) production were plotted to understand the manner in which the MOF membrane affects the catalytic operation (Figure 5 c,d, and Figure S8). All UiO‐66‐coated samples exhibited notably higher *j*_CO_ and rather similar *j* ${}_{H{}_{2}}$ in comparison to the bare Ag foil, thus hinting that the kinetics of electrocatalytic CO_2_ reduction to CO is accelerated by the presence of the MOF membrane.

Furthermore, the electrochemically active surface area (EASA) of all samples, measured by cyclic voltametric analysis in the non‐faradic region at different scan rates, was essentially unchanged (Figure S9, and Table S3). To monitor the chemical and electronic properties of the Ag electrocatalyst during the growth of the MOF, XPS measurements were performed for the UiO‐66‐(A‐D) coated Ag films by removing the MOF layer with an inert Ar‐ion gun. The Ag 3d_3/2_ and 3d_5/2_ peaks were not affected by the presence of the MOF membrane (Figure S10). This suggests that the synthetic condition of the UiO‐66 does not generate AgO on the UiO‐66‐Ag interface and thus not affect the chemical/electronic nature of the catalyst. Furthermore, to eliminate any ambiguity in the XPS measurement arising from the use of the Ar‐ion gun during etching of the UiO‐66‐(A‐D), a control experiment was performed with bare Ag electrode treated in an identical condition as that of the MOF synthesis (i.e., kept for 4 hours at 110 °C temperature in a closed container in presence of DMF + acetic acid vapor without using the precursor of the UiO‐66 MOF, termed Ag‐110 °C‐DMF). Since Ag‐110 °C‐DMF did not contain any MOF coating, the XPS measurement could be performed without any etching by Ar‐ion gun. A comparison of the XPS of Ag‐110 °C‐DMF and bare (unreacted) Ag, suggests no change of the oxidation‐state of the Ag (Figure S11). Furthermore, the XRD pattern of Ag‐110 °C‐DMF showed no additional peak of crystalline silver oxide (Figure S12). The Raman spectrum (Figure S13) of the Ag‐110 °C‐DMF was also similar to the one of bare Ag. All these results support the claim of no conversion of Ag to AgO during the synthesis of the UiO‐66 thin film. Hence, it is evident that the synthesis of the UiO‐66 did not change the chemical and electronic nature of the silver catalyst.

**Figure 6:**
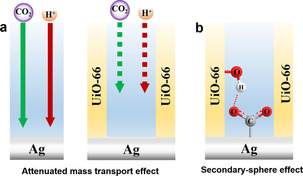
Illustration of a) attenuated mass transport effect and b) stabilization of reactive Ag‐bound *COO^−^ intermediate by secondary‐sphere interaction during CO_2_ electroreduction.

**Figure 7:**
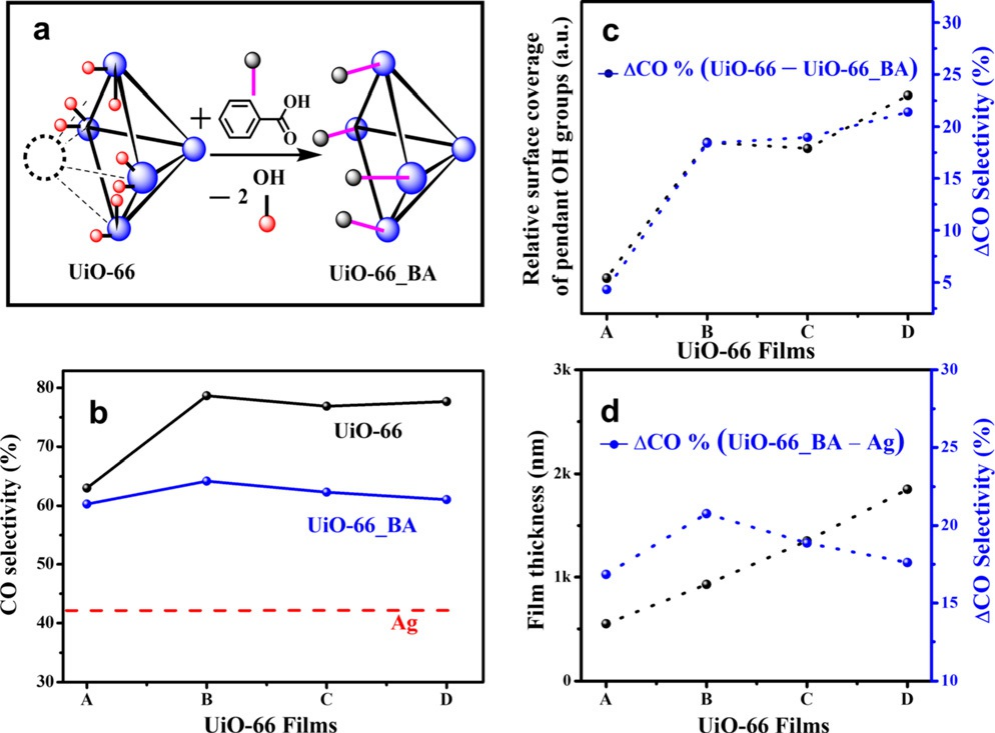
a) Schematic illustration of benzoic acid (BA) capping of UiO‐66 nodes via post‐synthetic modification. b) Variation of CO selectivity at −0.8 V vs. RHE, for the bare Ag, UiO‐66 and UiO‐66_BA electrodes. c) The variation in the relative surface coverage of pendant OH groups at the (UiO‐66–Ag) interface and the difference in CO selectivity, that is, ΔCO% (UiO‐66–UiO‐66_BA) for various UiO‐66 thin films. d) Thickness dependence of mass‐transport induced CO selectivity modulation, that is, ΔCO% (UiO‐66_BA–Ag).

As the properties of the active Ag electrocatalyst were not altered by the growth of the UiO‐66 membrane, other factors must dictate the remarkable increase in CO selectivity for UiO‐66‐(A‐D). As illustrated in Figure 6 a,b, two additional mechanisms could take place. (i) The porous UiO‐66 membrane could attenuate the mass transport of reactants towards the Ag electrocatalyst surface (Figure 6 a). In 0.1 M NaHCO_3_ electrolyte, the bulk concentration of dissolved CO_2_ (33 mM) is more than four orders of magnitude larger than the H^+^ concentration (1.6×10^−7^ M); in other words, although local concentration gradients will be generated for both species, compared to CO_2_, protons are more prone to be depleted at the catalyst‐electrolyte interface during catalytic operation, shifting the catalytic reaction towards CO_2_‐to‐CO conversion.[^16b^, ^17b^, ^21^]

(ii) The porous UiO‐66 membrane could modulate the secondary coordination sphere of the catalyst (secondary‐sphere effect, Figure 6 b) at the catalyst‐MOF interface. MC defects in UiO‐66 generate pendant OH groups at the Zr_6_‐oxo nodes, acting as Brønsted acidic sites at the vicinity of the Ag catalyst. In turn, these sites can act as proton relays to stabilize *COO^−^ intermediates and hence increase the rate of CO production. As such, decoupling the contribution of each of these mechanisms is difficult because both are expected to occur simultaneously during electrocatalysis. Yet, we have realized that by capping the pendant proton‐relaying OH groups at the Zr_6_‐oxo nodes, one would eliminate the secondary‐sphere effect of the UiO‐66 membrane, while still attenuating mass transport. Hence, the UiO‐66‐coated Ag electrodes were reacted with benzoic acid (BA) to cap the exposed OH groups at the MOF nodes (hereafter referred as UiO‐66_BA), as illustrated in Figure 7 a (for details, see Supporting Information Experimental Section).^[22]^ PXRD (Figure S14) and SEM (Figure S15) analysis confirm that the post‐synthetic modification with BA did not change the crystal structure and morphology of the resulting MOF. Raman spectroscopy was employed to verify the tethering of BA to the Zr_6_‐oxo nodes (Figure S16). The relative intensity of the two peaks appearing at 1448 cm^−1^ and 1431 cm^−1^ (OCO symmetric stretching of carboxylate linkers) gets modified due to comprehensive contribution of OCO stretching of bound BA.[^9a^, ^19c^] The BA loading in UiO‐66‐(A‐D)_BA were determined by ^1^H NMR analysis (Figure S17,18). As expected, the BA loading for all samples follows a similar trend as for the density of Zr_6_ nodes. Meaning, UiO‐66‐(B‐D)_BA exhibit comparable BA loading while UiO‐66‐A_BA's BA loading was lower. Additionally, the prominent decrease in the percentage of “Zr‐O‐H” species in the O1s XPS (Figure S19) indicates the capping of the exposed node‐bound OH groups by BA. In that manner, BA‐capped Zr_6_‐oxo nodes would lack the ability to stabilize the Ag‐bound *COO‐ intermediate by secondary‐sphere interaction. Consequently, comparing the CO selectivity of UiO‐66, UiO‐66_BA, and bare Ag should disclose the respective contribution of each of the two mechanisms governing the enhancement in electrocatalytic performance. As expected, for all MOF membranes thicknesses, the electro‐catalytic CO selectivity of UiO‐66_BA (measured at −0.8 V vs. RHE) was found to be considerably lower than that of UiO‐66, yet higher than bare Ag's selectivity (Figure 7 b). For each UiO‐66 thickness, the difference in CO selectivity between UiO‐66 and UiO‐66_BA, i.e., ΔCO% (UiO‐66—UiO‐66_BA), should represent the contribution of the pendant OH groups to the overall electrocatalytic performance of the system (Figure 7 c). Assuming this is correct, ΔCO% (UiO‐66—UiO‐66_BA) should strongly correlate with the relative coverage of pendant OH groups at the catalyst surface. As mentioned earlier, an increased concentration of MC defects induces an increase of exposed OH groups (missing linkers) at the remaining Zr_6_‐oxo clusters, albeit with a lower surface coverage of clusters, i.e., a low amount of Zr_6_‐oxo clusters and pendant groups in contact with the Ag. As such, the ratio of Zr_6_‐oxo clusters to pendant OH groups (i.e., the XPS signal ratio of Zr‐O‐Zr to Zr‐OH) should correlate with the relative surface coverage of pendant OH groups. Indeed, as seen in Figure 7 c, ΔCO% (UiO‐66—UiO‐66_BA) for the different samples is closely correlated with the relative surface coverage of pendant OH groups (calculated as the ratio of Zr‐O‐Zr to Zr‐OH). Hence, the effect of the attenuated mass‐transport mechanism was disclosed by the CO selectivity difference be‐tween UiO‐66_BA and bare Ag, i.e., ΔCO% (UiO‐66_BA—Ag), as seen in Figure 7 d. Clearly, all UiO‐66 thicknesses exhibit noticeable ΔCO% (UiO‐66_BA—Ag), reaching a maximum value of 21 % for UiO‐66‐B. Thus, it can be concluded that the MOF membrane‐induced attenuation of mass transport plays an important role in modulating the electrocatalytic selectivity of the system. Overall, due to the interplay of both mechanisms (stabilization of the *COO‐ intermediate by secondary‐sphere interactions, and attenuated mass transport), the maximum selectivity for CO was achieved using UiO‐66‐B, which possesses an optimum combination of pendant Brønsted acidic sites and membrane thickness.

Additionally, to demonstrate the generality of utilizing secondary‐sphere interactions to facilitate PCET processes, we used OH^−^ as an electrochemical probe.^[23]^ To do so, for all samples (UiO‐66‐(A‐D) and bare Ag), we compared the reversible electrochemical adsorption and desorption of OH^−^ (see detailed discussion in Supporting Information). The overpotential required for OH^−^ adsorption and desorption on all UiO‐66‐coated electrodes was found to be lower than for bare Ag (Figure S21, and Table S4). This result is an outcome of the stabilizing interactions between the MOF's pendant Brønsted acidic sites and Ag‐bound hydroxides, yielding an increased OH^−^ binding energy at the MOF‐modified Ag surface (Figure S22). Hence, this experiment provides yet another strong support to the argument of a secondary‐sphere stabilization of the *COO‐ intermediate during electrocatalytic CO_2_ reduction performed with the UiO‐66‐coated Ag electrodes.

To validate the generality of the MOF‐membrane concept, 2 sets of experiments were conducted. First, to understand if the effect UiO‐66 is not limited only to flat surfaces and can also influence the electrocatalytic performance of high surface area electrodes, Ag nanoparticles were grown within UiO‐66‐B (termed Ag@UiO‐66‐B; see Experimental Section in Supporting Information). As shown in Figure S23, higher catalytic current density, and comparable CO product selectivity as that of UiO‐66‐B could be achieved, thus pointing that the presented concept could indeed be applied to high surface area electrodes. Second, to verify the suitability of the MOF‐membrane approach for different types of electrocatalysts, UiO‐66 thin film was grown on Au (termed UiO‐66‐Au), a well‐known electrocatalyst for CO_2_ to CO conversion.[^21^, ^24^] As clearly seen in Figure S24, higher catalytic currents and better selectivity towards CO formation could be achieved for UiO‐66‐Au compared to bare Au over a wide potential range. In other words, the electrocatalytic CO_2_ reduction improvement by Ag@UiO‐66‐B and UiO‐66‐Au demonstrate the generality and flexibility of the MOF‐membrane notion, to facilitate PCET processes.

**Figure 8:**
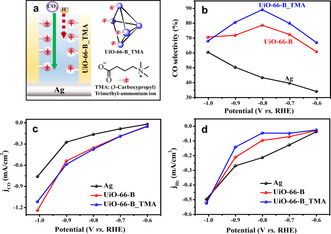
a) Illustration of a TMA ligand modified Zr_6_‐oxo node within a UiO‐66‐B_TMA electrode, and its effect on electrostatic ion‐gating of cationic species. b) Product selectivity, c) partial current density for the electroreduction of CO_2_ (*j*_CO_), and d) partial current density for hydrogen evolution reaction (*j* ${}_{H{}_{2}}$ ) of bare Ag, UiO‐66‐B, and UiO‐66‐B_TMA.

Next, we postulated that the concept of mass‐transport attenuation could be further extended by endowing the MOF membrane with permselectivity;^[25]^ tethering a ligand bearing fixed cationic charge within the MOF's pores should allow electrostatic tuning of cations (e.g., H^+^) ingress throughout the membrane towards the catalytically active sites, without affecting the permeability of neutral reactants, e.g., CO_2_ (see illustration in Figure 8 a).

To test this hypothesis, we modified the best performing sample (UiO‐66‐B) with 3(carboxypropyl)‐trimethyl‐ammonium (TMA; see Supporting Information Experimental Section for details). The resulting TMA‐modified MOF membrane (hereafter UiO‐66‐B_TMA) was characterized by PXRD, SEM and XPS (Figure S25–S28) and was found to retain the crystal structure and morphology characteristics of the parent UiO‐66‐B MOF. Confirmation of successful binding of TMA ligands to the MOF nodes was obtained by the recorded alteration of the relative intensity of UiO‐66‐B_TMA Raman peaks located at 1448 cm^−1^ and 1431 cm^−1^ as a result of contribution of symmetric OCO stretching of the TMA ligand bound to the Zr_6_‐oxo nodes (Figure S29).[^9a^, ^19c^] The loading level of the TMA was found to be 0.4 per Zr_6_‐oxo node, as determined by ^1^H NMR and ICP‐OES analysis (Figure S30, S31) (see detailed discussion in Supporting Information). We then compared the electrochemical response of UiO‐66‐B and UiO‐66‐B_TMA in the presence of a cationic redox probe, Ru(NH_3_)_6_^3+^ (Figure S32). Cyclic voltammograms recorded for UiO‐66‐B_TMA (Figure S32a) show a broadened, cathodically shifted reduction wave for Ru(NH_3_)_6_^3+^ compared to UiO‐66‐B, as expected for a sluggish electrochemical reaction due to the slower diffusion of the cationic species towards the Ag electrode surface.

In addition, reductive potential step chronoamperometry measurements were conducted to extract the Ru(NH_3_)_6_^3+^ diffusion coefficients for both electrodes (see detailed discussion in Supporting Information).^[9c]^ As seen in Table S5, the diffusion of Ru(NH_3_)_6_^3+^ within UiO‐66‐B_TMA is four times slower than in UiO‐66‐B, which confirms the ability to modulate the cation permeability in UiO‐66‐B_TMA.

With this knowledge in mind, we set to assess the electrocatalytic CO_2_ reduction performance of UiO‐66‐B_TMA (Figure 8 b–d). Remarkably, UiO‐66‐B_TMA exhibits enhanced CO selectivity over the tested potential range. At −0.8 V vs. RHE, CO selectivity reaches a maximum of 89 %, improving the catalytic selectivity of bare Ag and UiO‐66‐B by 46 % and 10 % respectively (Figure 8 b). Noticeably, the CO partial current densities for UiO‐66‐B_TMA and UiO‐66‐B are similar (Figure 8 c). Nevertheless, the H_2_ partial currents of UiO‐66‐B_TMA are significantly lower than those of UiO‐66‐B (Figure 8 d). Hence, it is apparent that while the rate of CO_2_ reduction to CO is not affected by the cation exclusion (ion‐gating) mechanism,^[26]^ the kinetics of H_2_ evolution is greatly suppressed due to the slower delivery of protons towards the active sites.

Finally, the stability of the best‐performing electrocatalytic system, UiO‐66‐B_TMA, was examined through a 5‐hour bulk‐electrolysis analysis conducted at −0.8 V vs. RHE. To counteract the low solubility of CO_2_ in aqueous NaHCO_3_ solution, we purged the solution with CO_2_ every hour for 30 mins (see Supporting Information Experimental Section). As seen in Figure S33, catalytic currents remained essentially constant over the entire period of measurement, while maintaining over 90 % of the initial CO selectivity. Additionally, as seen in Figure S34, XPS analysis of UiO‐66‐B_TMA before and after bulk‐electrolysis reveals at least a partial preservation of the MOF‐installed TMA ligand (as evident by the retained N1s peak). In addition, SEM images (Figure S35) and PXRD characterization (Figure S36) show a preserved MOF morphology and crystallinity upon electrolysis.

## Conclusion

In this work, we show that a non‐catalytic MOF membrane layered over a solid electrocatalyst, can systematically tune its catalytic performance at the molecular level, without alteration of the active site's properties. Specifically, as a proof of concept, a thin layer of UiO‐66 MOF was assembled on an Ag CO_2_ reduction electrocatalyst. It was found that the presence of the MOF overlayer leads to a substantial improvement in the system's electrocatalytic activity and selectivity towards CO production. The enhanced electrocatalysis was achieved as a result of three key mechanisms: i) the MOF acts as a porous membrane that attenuates mass transport of reactants towards the catalytic surface, generating local concentration gradients near the active sites, and hence modulates the catalytic reaction; ii) the stabilization of the reactive *COO^−^ intermediate by a secondary‐sphere interaction introduced by the MOF node's pendant Brønsted acidic groups positioned at the catalyst‐MOF interface; iii) the electrostatic ionic gating of protons: functionalization of MOF pores with fixed positively charged group enables control over delivery of H^+^ to the catalytic sites, thus improving the electrocatalytic selectivity. Importantly, the presented concept could in principle be extended for any given proton‐coupled electron transfer (PCET) reaction and hence provide a new, general tool kit for the design and manipulation of efficient heterogeneous electrocatalytic systems.

## Conflict of interest

The authors declare no conflict of interest.

## Supporting information

As a service to our authors and readers, this journal provides supporting information supplied by the authors. Such materials are peer reviewed and may be re‐organized for online delivery, but are not copy‐edited or typeset. Technical support issues arising from supporting information (other than missing files) should be addressed to the authors.

Supplementary

## References

[1a] H. Furukawa , K. E. Cordova , M. O'Keeffe , O. M. Yaghi , Science 2013, 341, 1230444; 23990564 10.1126/science.1230444

[1b] O. K. Farha , I. Eryazici , N. C. Jeong , B. G. Hauser , C. E. Wilmer , A. A. Sarjeant , R. Q. Snurr , S. T. Nguyen , A. Ö. Yazaydın , J. T. Hupp , J. Am. Chem. Soc. 2012, 134, 15016–15021; 22906112 10.1021/ja3055639

[1c] H. Furukawa , N. Ko , Y. B. Go , N. Aratani , S. B. Choi , E. Choi , A. Ö. Yazaydin , R. Q. Snurr , M. O'Keeffe , J. Kim , O. M. Yaghi , Science 2010, 329, 424. 20595583 10.1126/science.1192160

[2a] A. Phan , C. J. Doonan , F. J. Uribe-Romo , C. B. Knobler , M. O'Keeffe , O. M. Yaghi , Acc. Chem. Res. 2010, 43, 58–67; 19877580 10.1021/ar900116g

[2b] K. Sumida , D. L. Rogow , J. A. Mason , T. M. McDonald , E. D. Bloch , Z. R. Herm , T.-H. Bae , J. R. Long , Chem. Rev. 2012, 112, 724–781; 22204561 10.1021/cr2003272

[2c] Y. Peng , V. Krungleviciute , I. Eryazici , J. T. Hupp , O. K. Farha , T. Yildirim , J. Am. Chem. Soc. 2013, 135, 11887–11894. 23841800 10.1021/ja4045289

[3a] S. Bobbitt , M. L. Mendonca , A. J. Howarth , T. Islamoglu , J. T. Hupp , O. K. Farha , R. Q. Snurr , Chem. Soc. Rev. 2017, 46, 3357–3385; 28345694 10.1039/c7cs00108h

[3b] J.-R. Li , J. Sculley , H.-C. Zhou , Chem. Rev. 2012, 112, 869–932. 21978134 10.1021/cr200190s

[4a] P. Deria , D. A. Gómez-Gualdrón , I. Hod , R. Q. Snurr , J. T. Hupp , O. K. Farha , J. Am. Chem. Soc. 2016, 138, 14449–14457; 27768297 10.1021/jacs.6b09113

[4b] J. Katz , J. E. Mondloch , R. K. Totten , J. K. Park , S. T. Nguyen , O. K. Farha , J. T. Hupp , Angew. Chem. Int. Ed. 2014, 53, 497–501; 10.1002/anie.201307520 24273208

[4c] J.-Y. Lee , O. K. Farha , J. Roberts , K. A. Scheidt , S. T. Nguyen , J. T. Hupp , Chem. Soc. Rev. 2009, 38, 1450–1459; 19384447 10.1039/b807080f

[4d] J. Liu , L. Chen , H. Cui , J. Zhang , L. Zhang , C.-Y. Su , Chem. Soc. Rev. 2014, 43, 6011–6061; 24871268 10.1039/c4cs00094c

[4e] M. Zhao , S. Ou , C.-D. Wu , Acc. Chem. Res. 2014, 47, 1199–1207. 24499017 10.1021/ar400265x

[5a] C.-H. Chuang , C.-W. Kung , Electroanalysis 2020, 32, 1885–1895;

[5b] L. E. Kreno , K. Leong , O. K. Farha , M. Allendorf , R. P. Van Duyne , J. T. Hupp , Chem. Rev. 2012, 112, 1105–1125; 22070233 10.1021/cr200324t

[5c] Y.-C. Wang , Y.-C. Chen , W.-S. Chuang , J.-H. Li , Y.-S. Wang , C.-H. Chuang , C.-Y. Chen , C.-W. Kung , ACS Appl. Nano Mater. 2020, 3, 9440–9448.

[6a] S. Goswami , M. Chen , M. R. Wasielewski , O. K. Farha , J. T. Hupp , ACS Appl. Mater. Interfaces 2018, 10, 34409–34417; 30207679 10.1021/acsami.8b14977

[6b] K. Meyer , M. Ranocchiari , J. A. van Bokhoven , Energy Environ. Sci. 2015, 8, 1923–1937;

[6c] J. Yu , R. Anderson , X. Li , W. Xu , S. Goswami , S. S. Rajasree , K. Maindan , D. A. Gómez-Gualdrón , P. Deria , J. Am. Chem. Soc. 2020, 142, 11192–11202; 32449353 10.1021/jacs.0c03949

[6d] T. Zhang , W. Lin , Chem. Soc. Rev. 2014, 43, 5982–5993. 24769551 10.1039/c4cs00103f

[7a] S. Zhao , C. Tan , C.-T. He , P. An , F. Xie , S. Jiang , Y. Zhu , K.-H. Wu , B. Zhang , H. Li , J. Zhang , Y. Chen , S. Liu , J. Dong , Z. Tang , Nat. Energy 2020, 5, 881–890;

[7b] S. Zhao , Y. Wang , J. Dong , C.-T. He , H. Yin , P. An , K. Zhao , X. Zhang , C. Gao , L. Zhang , J. Lv , J. Wang , J. Zhang , A. M. Khattak , N. A. Khan , Z. Wei , J. Zhang , S. Liu , H. Zhao , Z. Tang , Nat. Energy 2016, 1, 16184.

[8] I. Hod , M. D. Sampson , P. Deria , C. P. Kubiak , O. K. Farha , J. T. Hupp , ACS Catal. 2015, 5, 6302–6309.

[9a] I. Liberman , R. Shimoni , R. Ifraemov , I. Rozenberg , C. Singh , I. Hod , J. Am. Chem. Soc. 2020, 142, 1933–1940; 31910614 10.1021/jacs.9b11355 PMC7467674

[9b] S. Mukhopadhyay , O. Basu , R. Nasani , S. K. Das , Chem. Commun. 2020, 56, 11735–11748; 10.1039/d0cc03659e 32940258

[9c] R. Shimoni , W. He , I. Liberman , I. Hod , J. Phys. Chem. C 2019, 123, 5531–5539.

[10a] N. Kornienko , Y. Zhao , C. S. Kley , C. Zhu , D. Kim , S. Lin , C. J. Chang , O. M. Yaghi , P. Yang , J. Am. Chem. Soc. 2015, 137, 14129–14135; 26509213 10.1021/jacs.5b08212

[10b] E. M. Miner , L. Wang , M. Dincă , Chem. Sci. 2018, 9, 6286–6291. 30123483 10.1039/c8sc02049c PMC6063138

[11a] P. M. Usov , B. Huffman , C. C. Epley , M. C. Kessinger , J. Zhu , W. A. Maza , A. J. Morris , ACS Appl. Mater. Interfaces 2017, 9, 33539–33543; 28353341 10.1021/acsami.7b01547

[11b] S. Lin , Y. Pineda-Galvan , W. A. Maza , C. C. Epley , J. Zhu , M. C. Kessinger , Y. Pushkar , A. J. Morris , ChemSusChem 2017, 10, 514–522. 27976525 10.1002/cssc.201601181

[12a] I. Hod , P. Deria , W. Bury , J. E. Mondloch , C.-W. Kung , M. So , M. D. Sampson , A. W. Peters , C. P. Kubiak , O. K. Farha , J. T. Hupp , Nat. Commun. 2015, 6, 8304; 26365764 10.1038/ncomms9304 PMC4647847

[12b] H. K. Lee , C. S. L. Koh , Y. H. Lee , C. Liu , I. Y. Phang , X. Han , C.-K. Tsung , X. Y. Ling , Sci. Adv. 2018, 4, eaar3208; 29536047 10.1126/sciadv.aar3208 PMC5844712

[12c] A. Wagner , C. D. Sahm , E. Reisner , Nat. Catal. 2020, 3, 775–786;

[12d] Y. Yang , S.-Q. Wang , H. Wen , T. Ye , J. Chen , C.-P. Li , M. Du , Angew. Chem. Int. Ed. 2019, 58, 15362–15366; 10.1002/anie.201909770 31441563

[13a] I. Bento , C. S. Silva , Z. Chen , L. O. Martins , P. F. Lindley , C. M. Soares , BMC Struct. Biol. 2010, 10, 28; 20822511 10.1186/1472-6807-10-28 PMC2944330

[13b] G. Brändén , R. B. Gennis , P. Brzezinski , Biochim. Biophys. Acta Bioenerg. 2006, 1757, 1052–1063; 10.1016/j.bbabio.2006.05.020 16824482

[13c] I. Hofacker , K. Schulten , Proteins: Struct. Funct. Genet. 1998, 30, 100–107; 9443344

[13d] K. Kirchberg , H. Michel , U. Alexiev , Biochim. Biophys. Acta Bioenerg. 2013, 1827, 276–284. 10.1016/j.bbabio.2012.10.014 23123516

[14] A. W. Nichols , C. W. Machan , Front. Chem. 2019, 7, 397. 31263689 10.3389/fchem.2019.00397 PMC6584898

[15a] Q. Gong , P. Ding , M. Xu , X. Zhu , M. Wang , J. Deng , Q. Ma , N. Han , Y. Zhu , J. Lu , Z. Feng , Y. Li , W. Zhou , Y. Li , Nat. Commun. 2019, 10, 2807; 31243275 10.1038/s41467-019-10819-4 PMC6594929

[15b] C. W. Lee , N. H. Cho , S. W. Im , M. S. Jee , Y. J. Hwang , B. K. Min , K. T. Nam , J. Mater. Chem. A 2018, 6, 14043–14057;

[15c] C. W. Lee , N. H. Cho , K. T. Nam , Y. J. Hwang , B. K. Min , Nat. Commun. 2019, 10, 3919; 31477719 10.1038/s41467-019-11903-5 PMC6718411

[15d] H. Yang , N. Han , J. Deng , J. Wu , Y. Wang , Y. Hu , P. Ding , Y. Li , Y. Li , J. Lu , Adv. Energy Mater. 2018, 8, 1801536;

[15e] B. A. Rosen , A. Salehi-Khojin , M. R. Thorson , W. Zhu , D. T. Whipple , P. J. A. Kenis , R. I. Masel , Science 2011, 334, 643–644; 21960532 10.1126/science.1209786

[15f] B. A. Rosen , J. L. Haan , P. Mukherjee , B. Braunschweig , W. Zhu , A. Salehi-Khojin , D. D. Dlott , R. I. Masel , J. Phys. Chem. C 2012, 116, 15307–15312;

[15g] Y. i. Hori , Modern aspects of electrochemistry, Springer, Heidelberg, 2008, pp. 89–189;

[15h] R. Kas , K. Yang , D. Bohra , R. Kortlever , T. Burdyny , W. A. Smith , Chem. Sci. 2020, 11, 1738; 34123269 10.1039/c9sc05375a PMC8150108

[15i] M. Ma , K. Liu , J. Shen , R. Kas , W. A. Smith , ACS Energy Lett. 2018, 3, 1301–1306; 29911182 10.1021/acsenergylett.8b00472 PMC5996346

[15j] X. Peng , S. G. Karakalos , W. E. Mustain , ACS Appl. Mater. Interfaces 2018, 10, 1734–1742. 29264918 10.1021/acsami.7b16164

[16a] S. A. Mahyoub , F. A. Qaraah , C. Chen , F. Zhang , S. Yan , Z. Cheng , Sustainable Energy Fuels 2020, 4, 50–67;

[16b] Y. Yoon , A. S. Hall , Y. Surendranath , Angew. Chem. Int. Ed. 2016, 55, 15282–15286; 10.1002/anie.201607942 27862743

[17a] D.-H. Nam , P. De Luna , A. Rosas-Hernández , A. Thevenon , F. Li , T. Agapie , J. C. Peters , O. Shekhah , M. Eddaoudi , E. H. Sargent , Nat. Mater. 2020, 19, 266–276; 32099112 10.1038/s41563-020-0610-2

[17b] D. L. T. Nguyen , C. W. Lee , J. Na , M.-C. Kim , N. D. K. Tu , S. Y. Lee , Y. J. Sa , D. H. Won , H.-S. Oh , H. Kim , B. K. Min , S. S. Han , U. Lee , Y. J. Hwang , ACS Catal. 2020, 10, 3222–3231.

[18] E. Virmani , J. M. Rotter , A. Mähringer , T. von Zons , A. Godt , T. Bein , S. Wuttke , D. D. Medina , J. Am. Chem. Soc. 2018, 140, 4812–4819. 29542320 10.1021/jacs.7b08174

[19a] M. J. Cliffe , W. Wan , X. Zou , P. A. Chater , A. K. Kleppe , M. G. Tucker , H. Wilhelm , N. P. Funnell , F.-X. Coudert , A. L. Goodwin , Nat. Commun. 2014, 5, 4176; 24946837 10.1038/ncomms5176 PMC4730551

[19b] G. C. Shearer , S. Chavan , S. Bordiga , S. Svelle , U. Olsbye , K. P. Lillerud , Chem. Mater. 2016, 28, 3749–3761;

[19c] C. Atzori , G. C. Shearer , L. Maschio , B. Civalleri , F. Bonino , C. Lamberti , S. Svelle , K. P. Lillerud , S. Bordiga , J. Phys. Chem. C 2017, 121, 9312–9324;

[19d] X. Feng , J. Hajek , H. S. Jena , G. Wang , S. K. P. Veerapandian , R. Morent , N. D. Geyter , K. Leyssens , A. E. J. Hoffman , V. Meynen , C. Marquez , D. E. D. Vos , V. V. Speybroeck , K. Leus , P. V. D. Voort , J. Am. Chem. Soc. 2020, 142, 3174–3183; 31971786 10.1021/jacs.9b13070 PMC7020139

[19e] B. Moll , T. Muller , C. Schlusener , A. Schmitz , P. B. S. Ozturk , C. Janiak , Mater. Adv. 2021, 2, 804.

[20a] A. Wang , Y. Zhou , Z. Wang , M. Chen , L. Sun , X. Liu , RSC Adv. 2016, 6, 3671–3679;

[20b] Y. Wang , L. Li , P. Dai , L. Yan , L. Cao , X. Gu , X. Zhao , J. Mater. Chem. A 2017, 5, 22372–22379.

[21] A. S. Hall , Y. Yoon , A. Wuttig , Y. Surendranath , J. Am. Chem. Soc. 2015, 137, 14834–14837. 26536054 10.1021/jacs.5b08259

[22] P. Deria , W. Bury , I. Hod , C.-W. Kung , O. Karagiaridi , J. T. Hupp , O. K. Farha , Inorg. Chem. 2015, 54, 2185–2192. 25665089 10.1021/ic502639v

[23a] X. Lu , T. Yu , H. Wang , L. Qian , P. Lei , ACS Catal. 2020, 10, 8860–8869;

[23b] A. Salehi-Khojin , H.-R. M. Jhong , B. A. Rosen , W. Zhu , S. Ma , P. J. A. Kenis , R. I. Masel , J. Phys. Chem. C 2013, 117, 1627–1632.

[24] A. Goyal , G. Marcandalli , V. A. Mints , M. T. M. Koper , J. Am. Chem. Soc. 2020, 142, 4154–4161. 32041410 10.1021/jacs.9b10061 PMC7059182

[25] I. Hod , W. Bury , D. M. Gardner , P. Deria , V. Roznyatovskiy , M. R. Wasielewski , O. K. Farha , J. T. Hupp , J. Phys. Chem. Lett. 2015, 6, 586–591. 26262471 10.1021/acs.jpclett.5b00019

[26] Note: We note that modification of MOF-nodes with TMA ligands replaces pendant hydroxide groups. Yet, the pendant positively charged ammonium functional group of TMA could also potentially participate in secondary-sphere interactions with the Ag-bound *COO^−^ intermediates. Thus, the effect of secondary-sphere interaction on CO selectivity of UiO-66-B_TMA should in principle be comparable to the one of UiO-66-B.

