# Supplementary material for: Assembly of a Metal–Organic Framework (MOF) Membrane on a Solid Electrocatalyst: Introducing Molecular‐Level Control Over Heterogeneous CO2 Reduction
Source: Angew Chem Int Ed Engl. 2021 May 5;60(24):13423–9. doi: 10.1002/anie.202102320 (PMC8251703; doi:10.1002/anie.202102320)
Supplement: Supplementary file 1 — Supplementary [file ANIE-60-13423-s001.pdf]

Angewandte Chemie
Eine Zeitschrift der Gesellschaft Deutscher Chemiker

Supporting Information

Assembly of a Metal–Organic Framework (MOF) Membrane on a Solid Electrocatalyst: Introducing Molecular-Level Control Over Heterogeneous CO_2 Reduction

*Subhabrata Mukhopadhyay, Ran Shimoni, Itamar Liberman, Raya Ifraemov, Illya Rozenberg, and Idan Hod**

anie_202102320_sm_miscellaneous_information.pdf

Supporting Information
Table of Contents

Section	Page Number
**Table of Contents**	S2
**Experimental procedures**	S3-S5
**Physical characterization methods**	S6
**Result and discussions**	S7-S27

Experimental Procedures

The chemicals.

Zirconyl dichloride octahydrate ($ZrOCl_2·8H_2O$ ≥99.5%), 1,4-benzenedicarboylic acid (BDC: $C_8H_6O_4$ ≥98%), (3-carboxypropyl)trimethylammonium chloride (TMA Chloride, $C_7H_{16}NO_2Cl$ ≥ 97%), trimesic acid ($C_9H_6O_6$ ≥ 95%) and hexaammineruthenium(III) chloride ($[Ru(NH_3)_6]Cl_3$, ≥ 98%), silver nitrate ($AgNO_3$, ≥ 98%) were purchased from Sigma-Aldrich. Acetic acid (AA, CH_4CO_2, ≥ 98%), dimethylformamide (DMF, C_3H_7NO), sodium hydroxide (NaOH, AR), sodium borohydride ($NaBH_4$, AR), acetonitrile (CH_3CN, AR) ethanol (C_2H_5OH, AR) and methanol (CH_4O, AR), were purchased from Bio-Lab. benzoic acid (BA, $C_7H_6O_2$, ≥ 98%) was purchased from Alfa Aesar. The Ag metal plates (≥ 99%) and Au metal plates (≥ 99%) were purchased from Holland Moran. CO_2, and Ar gas (O_2, ≥ 98%) were purchased from Maxima.

Pretreatment of Ag plate.

The Ag plates were cut into small pieces of size 2 cm^2. Each piece of Ag plate was initially cleaned by sonication in soap solution, milli-q (mq) water and isopropanol (AR), respectively. These Ag plates were electrochemically cleaned by performing chronoamperometry at high cathodic potential for 30 mins to remove surface-adsorbed impurities. The Ag electrodes/plates were again washed thoroughly with mq water and used for synthesis and/or electrochemical measurements. Before any electrochemical analysis, we made sure that only one face of the metallic Ag was exposed to the solution. The opposite face of the metallic Ag was covered with an insulating tape that shows no electrochemical activity within the potential window of our interest.

Synthesis of UiO-66 thin film.

Preparation of the precursors for various thickness UiO-66 film.

Two precursors solutions were first prepared by ultrasonication for 20 minutes. (1) $ZrOCl_2$ (18.6 mg, 0.057 mmol) and acetic acid (266 µl) were dissolved in 10 ml of DMF and (2) BDC (10.4 mg, 0.062 mmol) was dissolved in 10 ml DMF, separately. The two precursor solutions were mixed in equal volume with each other and drop-casted on an Ag plate. To obtain various thicknesses, i.e. UiO-66-A-D, different amounts of total volume precursor solution were drop-casted. Of note, the required amount of the precursor solution for the thicker films was beyond the holding capacity of the Ag plate. Thus, a two-step synthesis was performed for the thicker samples. Details are given provided in Table S1.

UiO-66 thin film formation *via* vapor-assisted conversion.

A glass bottle/vessel with a glass cap (Chemglass, Pyrex, 1680, CG-8122, 30 × 60mm) was used for the vapor-assisted conversion (VAC) synthesis of UiO-66 thin films on Ag. A mixture of 4.2 ml of DMF and 0.8 ml of acetic acid was introduced inside the glass bottle/vessel. Then, a 2-cm^2 Ag electrode was put on top of an inverted 20 mL glass vial that was kept inside the glass bottle. The precursor solution of UiO-66 was drop-casted on the Ag electrode. To obtain various thicknesses of the UiO-66 thin film, different amounts of precursor solution were used (as mentioned before). For example, 300 µl of precursor solution was used for the synthesis of UiO-66-A thin film on Ag electrode. Then the glass bottle/vessel was capped and sealed using Teflon tape before keeping the whole setup at 110 ℃ for 4 hours. The thicker UiO-66 films (i.e. UiO-66-B-D) were prepared following a two-step synthesis, where the first step is identical to the synthesis of UiO-66-A. In the second step, the additional amount of the precursor solution was drop-casted on the already formed UiO-66 layer; for example, 150 µl of precursor solution were used in the second step for the synthesis of the UiO-66-B. After the synthesis, the Ag electrodes coated with the UiO-66 thin film were kept at 70 ℃ for 12 hours under vacuum. To prepare the UiO-66-Au, a similar procedure was followed as that of UiO-66-B (on Ag) while flat Au plate was used instead of flat Ag to grow the MOF.

Post-synthetic modification of UiO-66-B with TMA.

20 mg (0.11 mmol) of TMA was dissolved in 20 ml of methanol in a screw capped glass vial. The UiO-66-B film coated Ag electrode was dipped inside the solution. The vial was closed and sealed with Teflon before moving into a pre-heated oven kept at 50 ℃. The solution was kept at 50 ℃ for 24 hours. After completion, the electrode was washed by immersion in 30 ml of fresh methanol at 50 ℃ for 24 hours. Every 4 hours, the liquid was replaced with fresh methanol. Finally, the post-synthetically modified UiO-66-B_TMA electrode was dried in a vacuum oven for 3 hours.

Post-synthetic modification of UiO-66 thin films with BA.

Four screw-cap glass vials were filled with solution containing 50 mg (4.01 mmol) of BA dissolved in 20 ml of DMF. The vials were closed and sealed with Teflon before moving into a pre-heated oven kept at 80 ℃. Each vial was kept at 80 ℃ for 24 hours. After completion, the electrodes were washed by immersion in 30 ml of fresh DMF for 24 hours at the same temperature. Every 4 hours, the liquid was replaced with fresh DMF. Finally, the UiO-66_BA electrodes were dried in vacuum oven for 12 hours.

Post-synthetic incorporation of Ag nanoparticles to prepare the Ag@UiO-66-B.

The already prepared UiO-66-B (on Ag electrode) samples were dipped into a solution prepared of silver nitrate in acetonitrile (concentration: 0.5 mg/ml) for 48 hours. The Ag^+ soaked UiO-66-B was taken out and dried in a vacuum at 60 °C for 24 hours. Next the electrodes were dipped for 6 hours in 0.2 mmol/ml sodium borohydride ($NaBH_4$) prepared in ethanol. Following thorough wash of the electrode surface with water and ethanol, the electrodes were dried in a vacuum oven overnight to obtain the Ag@UiO-66-B.

Preparation of Ag-110°C-DMF.

As a control experiment to understand the probability of generation of silver oxide during the synthesis of the UiO-66 MOF on Ag electrode, Ag-110 °C-DMF was prepared. The bare Ag electrode was kept in similar synthetic condition as that of UiO-66. Meaning, the bare Ag plate was coated with DMF and acetic acid (in the same ratio as that of the precursor solution mixture of UiO-66 thin film) and kept at 110 °C for 4 hours on an inverted 20 ml glass vial that was kept inside a closed-cap glass bottle/vessel containing a mixture of 4.2 ml of DMF and 0.8 ml of acetic acid at the bottom. Basically, the complete synthetic condition of UiO-66 thin films on Ag electrode was replicated to prepare the Ag-110 °C-DMF sample, only without the MOF precursors.

Experimental methods for electrochemical characterization and catalysis.

A two-compartment cell using a three-electrode set up was used for the electrochemical measurements. One compartment contained the working and reference electrode while the counter electrode was placed in the other compartment. The two compartments were separated by a Nafion 117 membrane. Unless mentioned otherwise, a bare or UiO-66-coated Ag electrode was used as the working electrode. An Ag/AgCl electrode and a Pt-flag electrode were used as the reference and counter electrode, respectively. All electrochemical experiments were performed at ambient temperature and electrode potentials were converted to the RHE scale using the relation $E(RHE) = [E(Ag^+/Ag) – (0.059×pH) +0.197\ V]$. Most of the cyclic voltammograms and linear scan voltametric measurements were recorded at a 100 mVs^{-1} scan rate. The data was recorded with a Bio-Logic VSP instrument. A 0.1 M (aqueous) sodium bicarbonate solution was used as the electrolyte for most of the electrochemical measurements unless mentioned otherwise.

Electrochemical CO_2 reduction reaction (CRR) measurements: All electrochemical CRRs were performed in gas-tight, CO_2-saturated, two-compartment cell in a three-electrode configuration, with 0.1 M $NaHCO_3$ as the electrolyte (pH 6.8). Bare or UiO-66-coated Ag electrodes were used as the working electrode. An Ag/AgCl electrode and a Pt-flag electrode were used as the reference and counter electrode, respectively. The working compartment and the counter compartment were separated from each other by an ion conducting Nafion 117 membrane. The cathodic compartment, i.e. the compartment containing the working electrode, was filled with 40 ml of 0.1 M

$NaHCO_3$ solution for all the CRR measurements to maintain a constant headspace volume for all measurements. Before the measurement, both the compartments were purged with CO_2 for 1 hour and the cell was then sealed to maintain the CO_2 saturation. During the CRR, the electrolyte solution in the working compartment was stirred at a constant rate of 560 r.p.m. A set of different potentials were applied on each of the working electrodes for different time intervals, so that a constant charge of 1 C can be passed through the working electrode during each chronoamperometric CRR measurement. The gaseous products were collected from the sealed headspace with a VICI Pressure Lok Precision Analytical gas syringe and analyzed by gas chromatography (Perkin Elmer). Faradaic efficiencies were calculated using the formula:

Faradaic efficiency (FE) = nF × (m/Q)

Where, F= Faraday constant, n = number of electrons involved in the reaction, m = number of moles of product formed, Q = total charge passed = 1C. Thus, the modified equation FE-CO = (2× 96500C × (m-CO / 1 C)), where m-CO is the number of moles of CO.

Similarly, the modified equation FE-H_2 = (2× 96500C × (m-H_2 / 1 C)), where m-H_2 is the number of moles of H_2.

OH^- sorption-desorption experiment.

The adsorption and desorption of hydroxide was measured for the bare Ag and for the UiO-66-coated Ag surfaces. For the experiment, 0.1 M aqueous $NaClO_4$ was used as the supporting electrolyte and the pH was adjusted to 9 using 0.1 M NaOH. A one-compartment cell was used with a conventional three-electrode setup for the measurement. Bare and UiO-66-coated Ag electrodes were used as the working electrode in separate measurements. An Ag/AgCl electrode and a Pt-flag electrode were used as the reference and counter electrode respectively. Cyclic voltammograms were recorded within the potential window of 0.1 V to 0.8V (vs. Ag^+/Ag as reference electrode) with a scan rate of 20 mV/s.

$[Ru(NH_3)_6]^{3+}$ sorption-desorption experiment.

The adsorption of $[Ru(NH_3)_6]^{3+}$ was measured for the bare and UiO-66-B_TMA-thin film coated Ag surface. For the experiment, 0.1 M aqueous $NaClO_4$ was used as the supporting electrolyte. A one-compartment cell containing an Ar-saturated aqueous solution was used. The aqueous solution consisted of 100 mM of $[Ru(NH_3)_6]Cl_3$ in 0.1 M $NaClO_4$. The scan rate was -100 mV/s. A conventional three-electrode setup was employed for the measurements. Bare and UiO-66-B_TMA-coated Ag electrodes were used as the working electrode in separate measurements. An Ag/AgCl electrode and a Pt-flag electrode were used as the reference and counter electrode respectively. Cyclic voltammograms were recorded within the potential window of 0.05 V to -0.8V (vs. Ag^+/Ag as reference electrode) with a scan rate of -100 mV/s.

Furthermore, we were interested in comparing the $[Ru^{III}(NH_3)_6]^{3+}$ transport kinetics in UiO-66-B and UiO-66-B_TMA. Potential-step chronoamperometric measurements were carried out to determine the diffusion coefficient (D) of the $[Ru^{III}(NH_3)_6]^{3+}$ cationic probe in the two cases. We stepped the potential from fully oxidizing (0.1 V vs Ag^+/Ag, Ru^{3+} state in all $[Ru^{III}(NH_3)_6]^{3+}$) to fully reducing (−0.8 V vs Ag^+/Ag, Ru^{2+} state in all $[Ru^{III}(NH_3)_6]^{2+}$), while recording the current decay over time.

CRR stability measurement.

A CRR stability measurement was performed with an Ag modified with UiO-66-B_TMA as the working electrode. A CO_2-saturated 0.1 M $NaHCO_3$ solution was used as the electrolyte solution. A conventional three electrode set-up was used in a two-compartment cell, where the compartments are connected to each other by an ion-conducting Nafion membrane. For the chronoamperometric experiment, a potential of -0.8008 V (vs. RHE) was applied for five cycles of 60 min each. In between each measurement the electrolyte solution of both the compartments were purged with CO_2 for 30 minutes to maintain the CO_2 saturation in the solution. The amount of evolved CO and H_2 was monitored by gas chromatography at the end of each cycle.

Physical characterization methods.

The crystalline structures of the UiO-66 thin films were confirmed by powder X-ray diffraction (PXRD) using PAN analytical's Empyream multi-purpose diffractometer instrument and Cu-Kα (0.15405 nm) radiation.

The scanning electron microscope (SEM) images were taken using a Verios XHR 460L SEM instrument. Top view SEM images were recorded after performing carbon coating (5 nm) on the Ag electrodes modified with a UiO-66 thin film. Images were recorded at different magnifications.

The SEM-FIB (scanning electron microscopy with focused ion beam) images were recorded using a Thermo Scientific Dual-Beam system, in which the ion beam and the electron intersect each other at a 52° angle near the sample surface. The samples were first coated with a relatively thick layer of Au (30 nm) and then analyzed by SEM-FIB. During the analysis, the area under investigation was coated with 150 nm of Pt and on top of that with 1500 nm of carbon by ion beam assisted chemical vapor deposition. A small area was then cut by the sputtering of the focused ion beam to visualize the cross-sectional thickness of the membrane.

Quantachrome Autosorb IQ2 at 77 K was used to measre the N_2 physisorption isotherms for the UIO-66-(A-D). Each of the samples were degassed at 140°C in vacuum for 12 h prior to recording the isotherm. The surface area of the samples was calculated by fitting the isotherm data point of the absorption branch to Brunauer-Emmett-Teller (BET) model and the pore size distribution was determined by applying the NLDFT model to the isotherms of each sample.

To determine the amount of Zr in UiO-66-(A-D)_BA and UiO-66-B_TMA, ICP-OES analysis was performed using a Spectro ARCOS ICP-OES, FHX22 multi-View plasma (SOP, EOP) instrument. To prepare the sample, each UiO-66 sample of measured amount was digested in 5 ml of conc. HNO_3 at 150 ℃ for 12 hours. 1 ml of the acid solution was diluted to 10 ml for ICP-OES measurement.

Raman spectroscopy measurements were done in Horiba LabRam HR evolution micro-Raman system, equipped with a Synapse Open Electrode CCD detector, air cooled to -60 °C. The excitation source was a 532 nm laser and it was focused with an x50 objective lens. For the UiO-66-B and UiO-66-B_TMA directly the corresponding UiO-66 coated Ag plates were placed under the laser.

The X-ray photoelectron spectra were collected using an ESCALAB 250 apparatus X-ray photoelectron spectrometer equipped with an Al-Kα X-ray source and a monochromator. The survey spectra were recorded with a pass energy (PE) of 150 eV and the high energy resolution was achieved with a PE of 20 eV. To correct the charging effect, all measured spectra were calibrated relatively to the C 1s peak position at 284.6 eV. Furthermore, the samples were treated with an EX05 inert ion gas gun fitted in this instrument to etch away surface layers of UiO-66 for composition analysis of the Ag surface below the MOF, which is the active catalytic surface for CO_2 reduction. The XPSPEAK software (Version 4.1) was used for further analysis the XPS spectra of the samples. The data processing was done with the AVANTGE and XPSPEAK (version 4.1) programs.

The Hydrogen Nuclear Magnetic Resonance (H-NMR) measurements were carried out on a Bruker DPX-500 instrument. To evaluate the concentration of the benzoic acid (BA) in the UiO-66-(A-D)_BA, a calibration curve of BA was first prepared using different solutions of known concentration of BA in 2 M NaOH (in D_2O). The different UiO-66 samples were dissolved in known amount in 2 M NaOH (in D_2O). Using the calibration plot the amount of the BA was calculated for each case. To evaluate the concentration of TMA in UiO-66-B_TMA, another calibration curve of TMA was prepared. The different concentration solutions of TMA were prepared in 2M NaOH/D_2O in a similar way as that was done for BA. Known amount of trimesic acid was used as internal standard for each NMR measurement. Known amount of UiO-66-B_TMA was dissolved in 2 M NaOH/D_2O to study the amount of the TMA loading.

The electrochemical measurements were performed using a BioLogic VSP-128 electrochemical workstation. Gas chromatography (GC) was performed using a PerkinElmer Clarus 590 GC equipped with a wide-range flame ionization detector (FID) and a thermal conductivity detector (TCD). The head-space gas (after electrolysis) was characterized and quantified by GC measurements. Measurements were performed by manual injection of 500 µl of head-space gas mixture using a Pressure Lok Precession Analytical Syringe.

Results and Discussions.

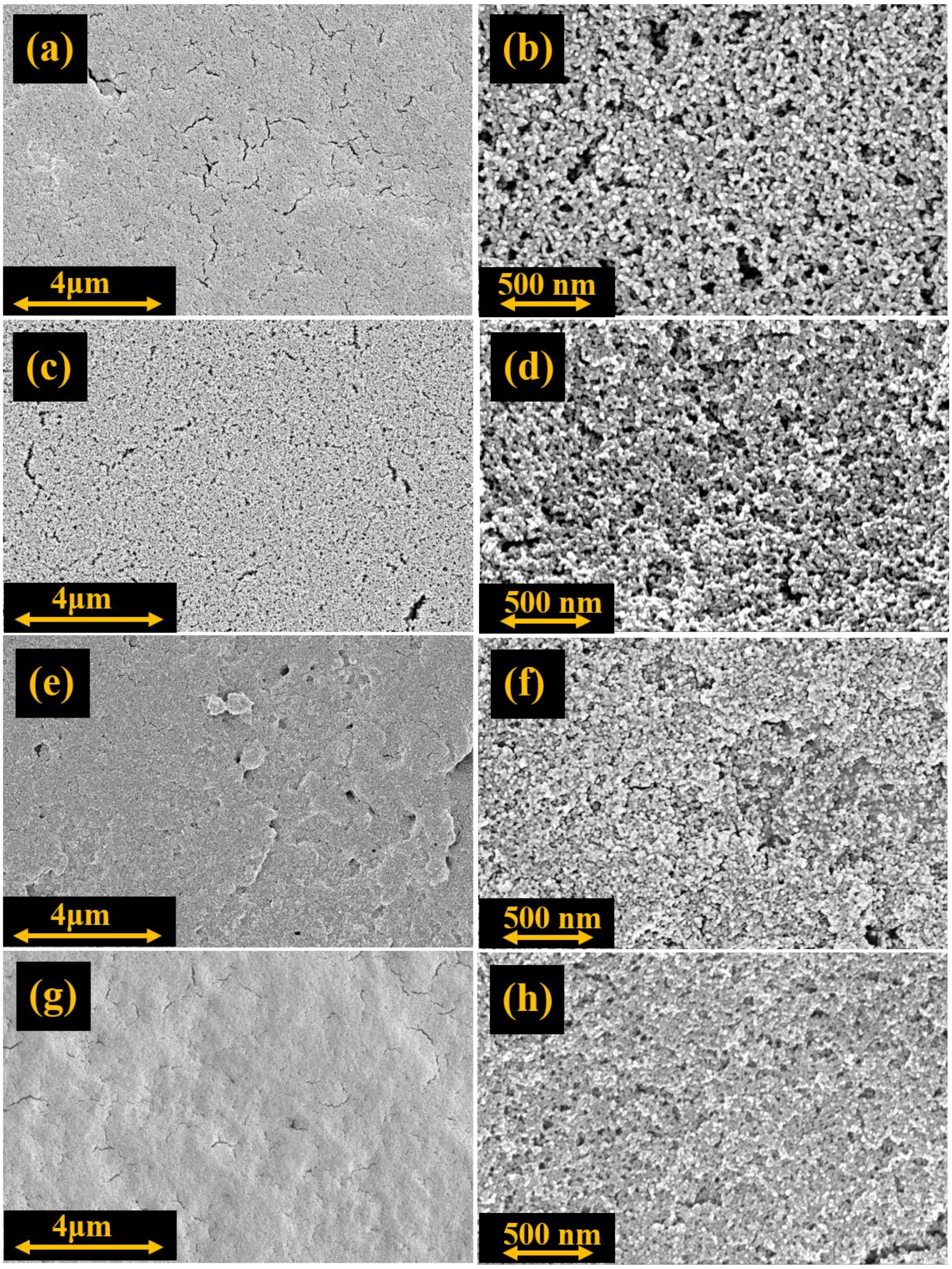

Figure S1. SEM images (top view) of (a-b) UiO-66-A, (c-d) UiO-66-B, (e-f) UiO-66-C and (g-h) UiO-66-D.

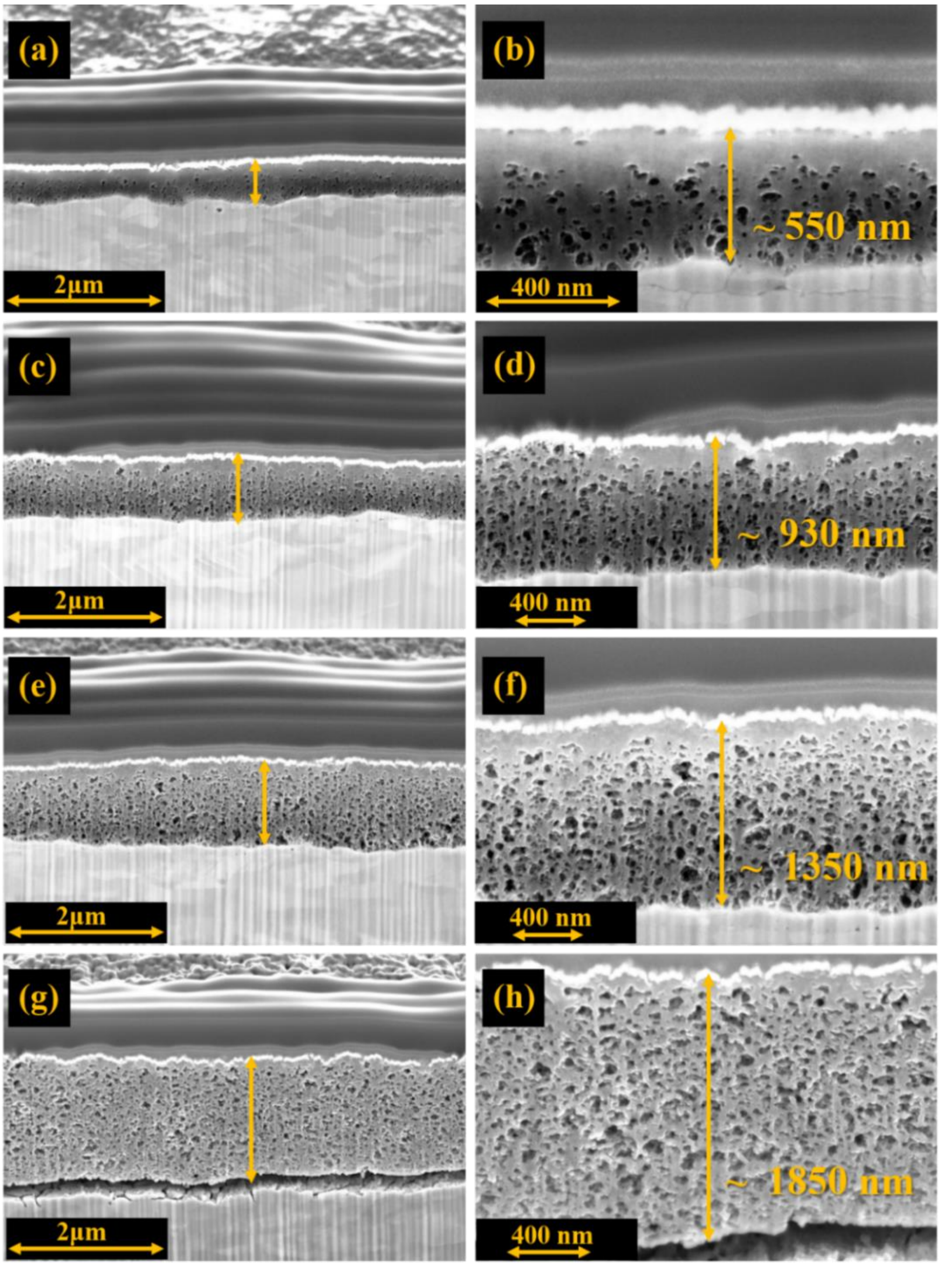

Figure S2. SEM-FIB cross section images of (a-b) UiO-66-A, (c-d) UiO-66-B, (e-f) UiO-66-C and (g-h) UiO-66-D.

Table S1. Details of synthetic condition for the preparation of UiO-66 thin films.

Sample code	Amount of total precursor solution	Number of steps of drop-casting	Total Reaction time
UiO-66-A	300 μl	One	4 hours
UiO-66-B	450 μl	Two	8 hours
UiO-66-C	525 μl	Two	8 hours
UiO-66-D	600 μl	Two	8 hours

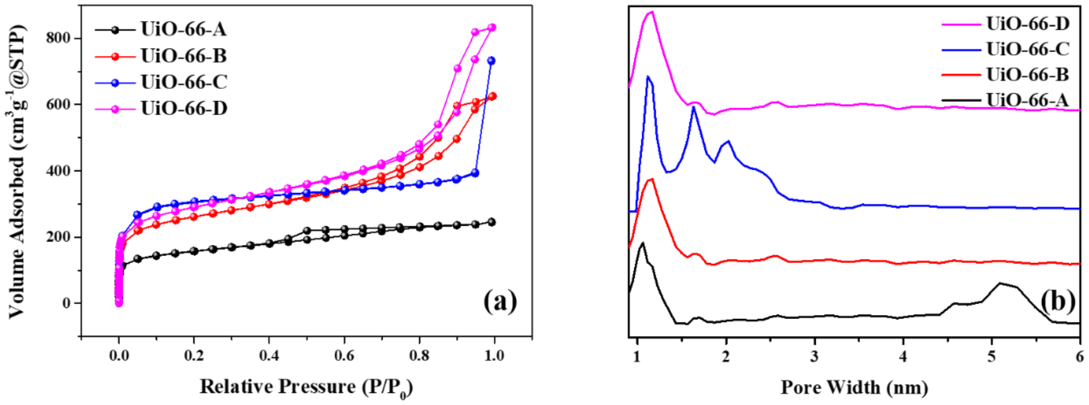

Figure S3. a) N_2 physisorption isotherms of the UiO-66-(A-D); b) the pore size distribution for the UiO-66-(A-D).

N_2 physisorption measurements reveal the BET surface area and the pore size distribution of UiO-66-(A-D). The pore size distribution shows the presence of a significant amount of large micropores (15 Å - 20 Å) in most of the samples which is expected because of the high defect density of the UiO-66-(A-D) thin films. Of note, the variation in pore size distribution and BET surface area of UiO-66-(B-D) cannot explain the relative order mass transport in the MOF thin films UiO-66-(A-D); because it is governed by both the factors: (a) thickness of the MOF film and (b) the pores size distribution of the MOF.

Table S2. BET surface area of UiO-66-(A-D)

**Sample**	**BET surface area (m^2g^{-1})**
**UiO-66-A**	567.83
**UiO-66-B**	945.80
**UiO-66-C**	1155.74
**UiO-66-D**	1046.82

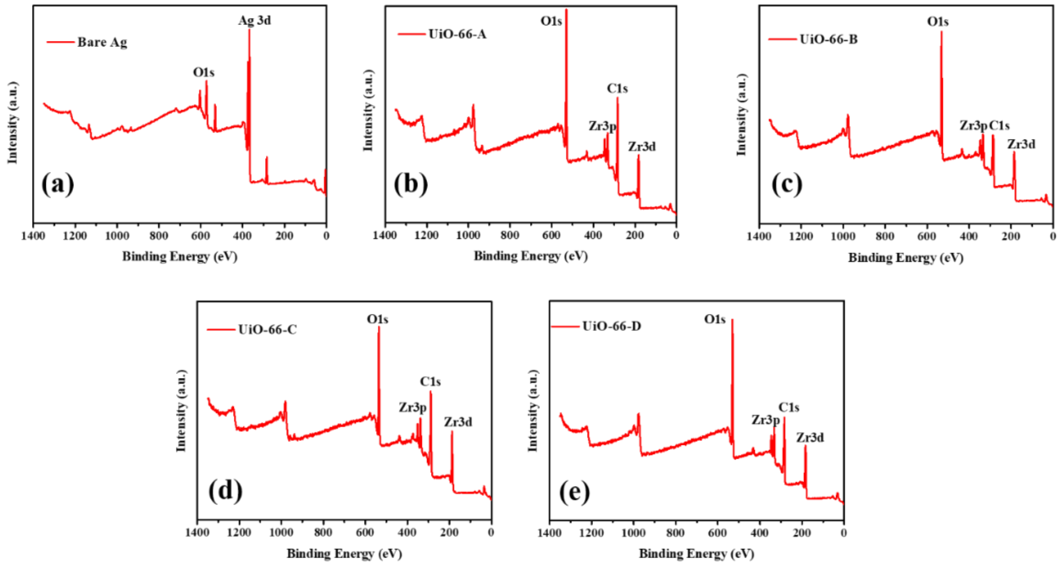

Figure S4. XPS survey scans of (a) bare Ag, (b) UiO-66-A, (c) UiO-66-B, (d) UiO-66-C and (e) UiO-66-D.

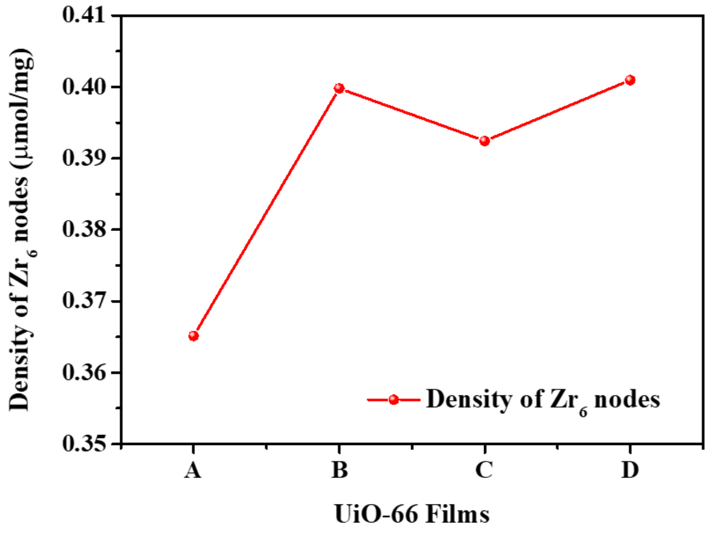

Figure S5. Density of Zr_6 nodes of UiO-66-(A-D) depicting the relative order of metal cluster (MC) defect.

Determination of density of Zr_6 nodes in UiO-66-(A-D) and comparison of metal cluster defect density:

The density of Zr_6 nodes for each of the UiO-66-(A-D) were determined from the ICP-OES measurement. For ICP-OES analysis, known amount of UiO-66-(A-D) were digested in 5 ml of conc. HNO_3 at 150 ℃ for 12 hours. 1 ml of the acid mixture was diluted to 10 ml by adding water and analyzed by ICP-OES.

From the experimentally obtained concentration of Zr ions in the 10 ml solution prepared from each UiO-66 MOF, the concentration of Zr_6 nodes were calculated. For example, 2.72 mg of UiO-66-A was digested in 5 ml of conc. HNO_3. Out of total 5 ml, 1 ml was diluted with water to 10 ml for ICP-OES measurement.

According to ICP-OES analysis, the concentration of Zr^{4+} in the solution for UiO-66-A was 10.872 × 10^{-6} mg/l. Thus, the concentration of Zr_6 (Formula weight of Zr_6 is 547.344) was calculated to be 0.365 µmol/mg for UiO-66-A.

In a defect free UiO-66 (Molecular formula $Zr_6O_4(OH)_4(OOC\text{-}C_6H_4\text{-}COO)_6$; molar mass 1664.06 g/mole) the density of Zr_6 should be ideally 0.614 µmol/mg. In case of the UiO-66-(A-D) the density of Zr_6 metal node is much lower than this ideal value. This suggests the high extent of MC defects in the UiO-66-(A-D) thin films, which result into lowering of Zr_6 density in case of the UiO-66 thin films. The extent of missing cluster (MC) defect of the UiO-66-(A-D) follows a similar trend as that of $[(Zr_6)_{ideal} – (Zr_6)_{UiO\text{-}(A\text{-}D)}]$ and similar trend has also been observed from the XPS and XRD analysis.

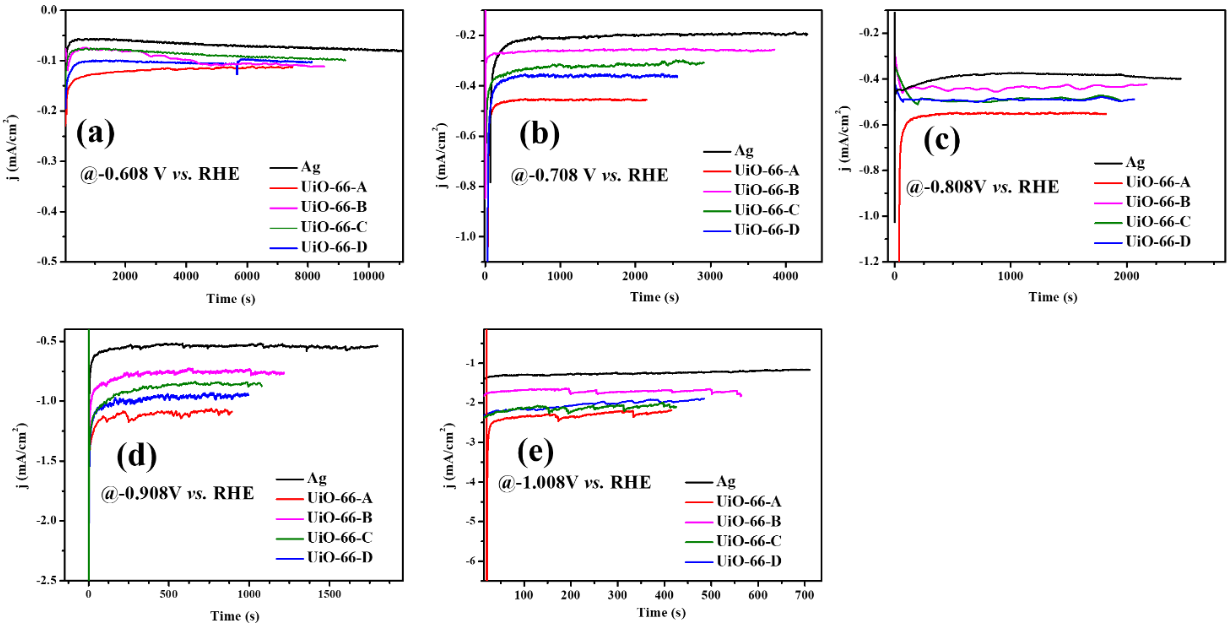

Figure S6. Chronoamperometric measurements conducted at a constant applied potential of (a) -0.6 V vs RHE, (b) -0.7 V vs RHE, (c) -0.8 V vs RHE, (d) -0.9 V vs RHE and (e) -1.0 V vs RHE.

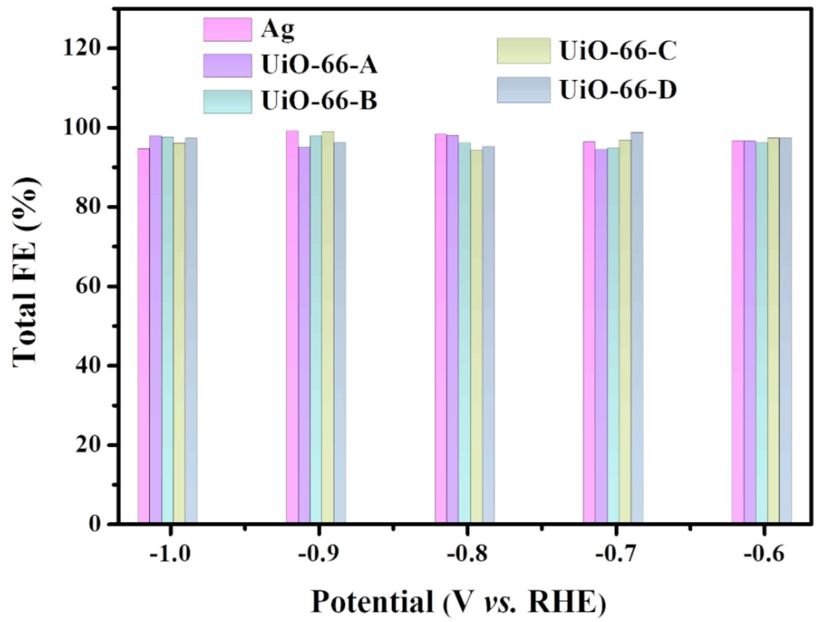

Figure S7. Total faradaic efficiencies ($CO + H_2$) for UiO-66-(A-D) and bare Ag. Electrolysis performed in the potential range -0.6V to -1.0V (vs. RHE) in CO_2 saturated 0.1 M $NaHCO_3$. Total charge passed in each case is 1C.

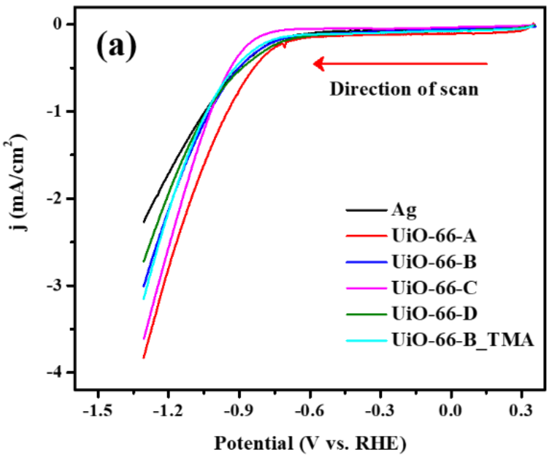

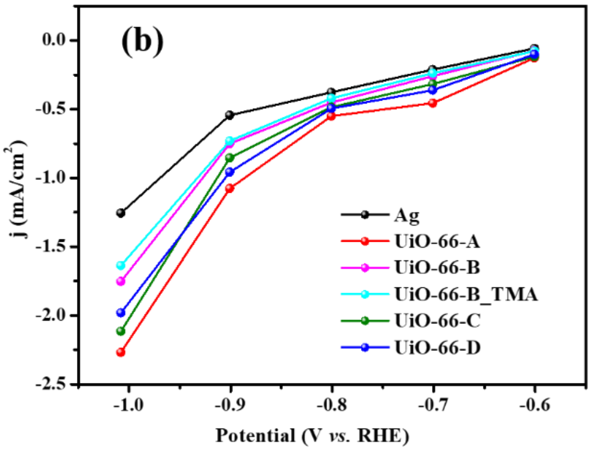

Figure S8. (a) Linear scan voltammograms and (b) steady state catalytic current of bare Ag, UiO-66-(A-D) and UiO-66-B_TMA. All measurements were done in CO_2-saturated 0.1 M $NaHCO_3$. Scan rate: 100 mV/s.

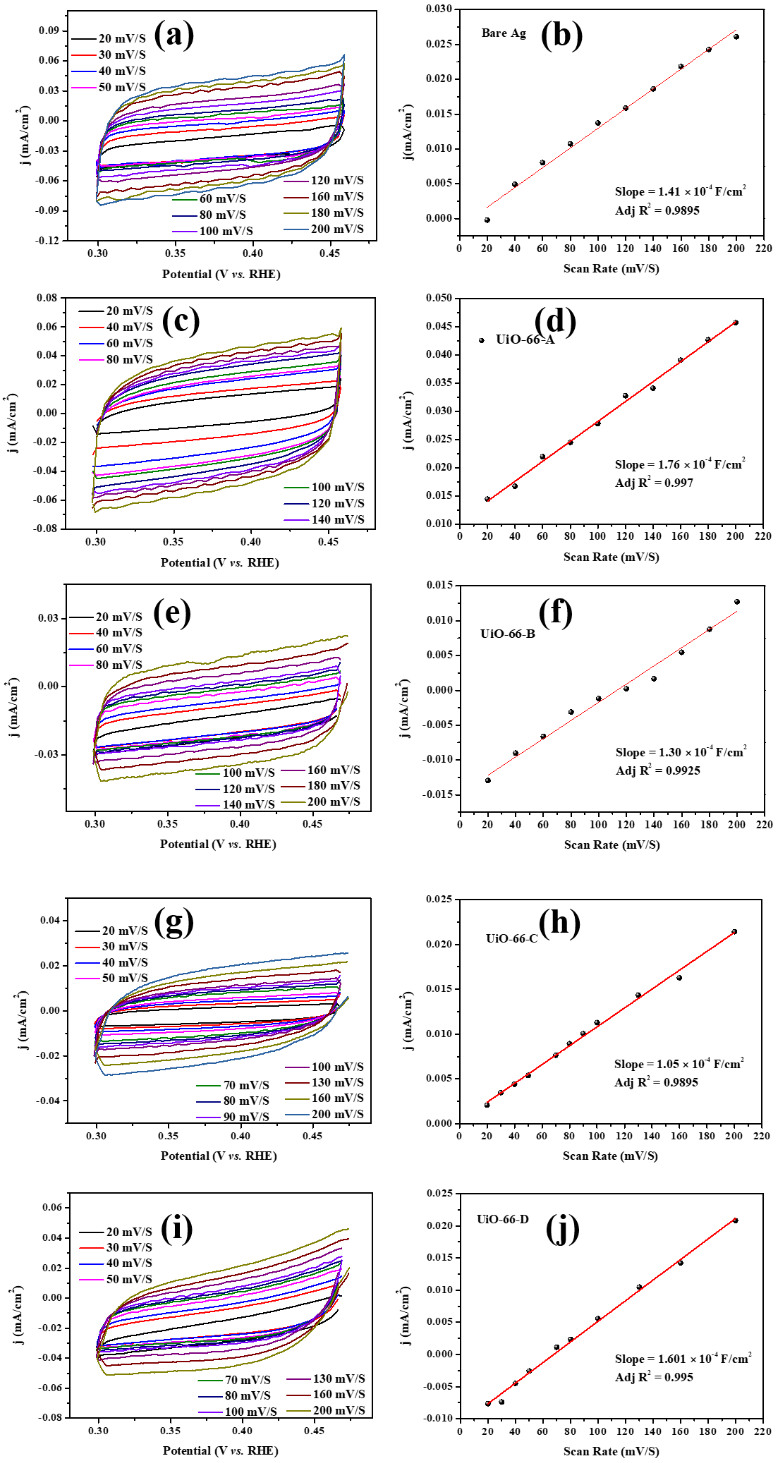

Figure S9. (a), (c), (e), (g), (i) Cyclic voltammograms recorded in the non-faradic region with different scan rates for bare Ag UiO-66-A, UiO-66-B, UiO-66-C and UiO-66-D, respectively. (b), (d), (f), (h), (j) linear fits for the anodic current @ 0.39 V (*vs.* RHE) for bare Ag, UiO-66-A, UiO-66-B, UiO-66-C and UiO-66-D, respectively.

Electrochemical surface area calculation.

The electrochemically active surface area (EASA) of an electrode increases with an increase in the double layer charge capacitance (C_{dl}). Thus, the trend of the EASA of the bare Ag electrode and the UiO-66-(A-D) coated Ag electrodes can be understood from their respective C_{dl} values. From the cyclic voltammograms recorded at different scan rates in the non-faradic region using 0.1 M $NaHCO_3$ solutions, the C_{dl} values of bare Ag and UiO-66-(A-D) thin film coated Ag electrodes can be determined (Figure S8. (a), (c), (e), (g), (i)). This involves the logical assumption that double layer charging is the only process taking place in this potential range. The slope of the straight-line section of the capacitive current vs. scan rate gives the double layer capacitance (C_{dl}). The slope of the straight line for each case is provided below in a tabular form (from Figure S8. (b), (d), (f), (h), (j)).

Table S3. Slope corresponding to C_{dl} of bare Ag and UiO-66-(A-D) thin film coated Ag electrodes

Sample	Slope of linear fit (Fcm^{-2})
Ag	1.41×10^{-4}
UiO-66-A	1.76×10^{-4}
UiO-66-B	1.30×10^{-4}
UiO-66-C	1.05×10^{-4}
UiO-66-D	1.60×10^{-4}

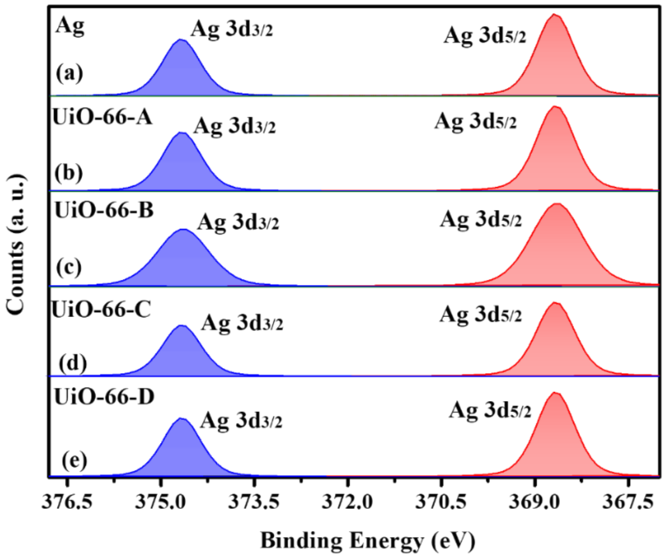

Figure S10. Ag 3d X-ray photoelectron spectra of (a) Ag (b) UiO-66-A (c) UiO-66-B (d) UiO-66-C and (e) UiO-66-D. The spectra were recorded after etching of the MOF coating by an EX05 inert ion gas gun.

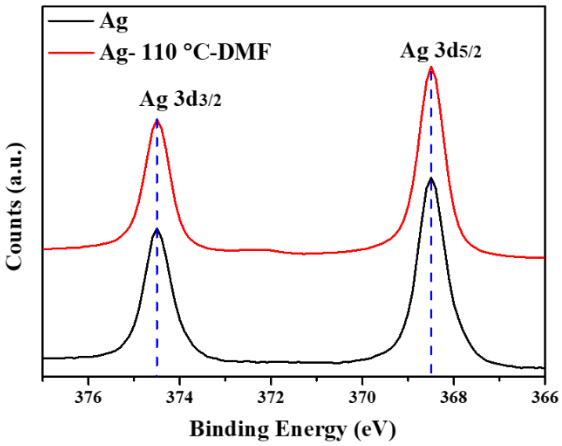

Figure S11. Ag 3d X-ray photoelectron spectra of Ag- 110 °C-DMF and bare Ag. To prepare Ag- 110 °C-DMF, Ag plate was treated with DMF and Acetic acid at 110 °C for 4 hours without the MOF precursors. Both the spectra were recorded without any etching by Ar-ion gun.

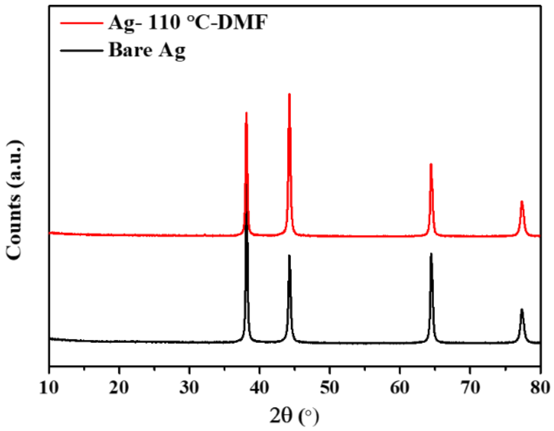

Figure S12. Comparison of XRD pattern of Ag-110°C-DMF and bare Ag.

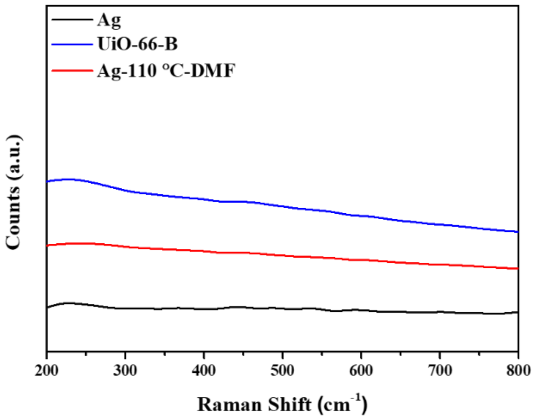

Figure S13. Comparison of Raman spectral features of Ag-110°C-DMF and bare Ag. The excitation source was a 532 nm laser.

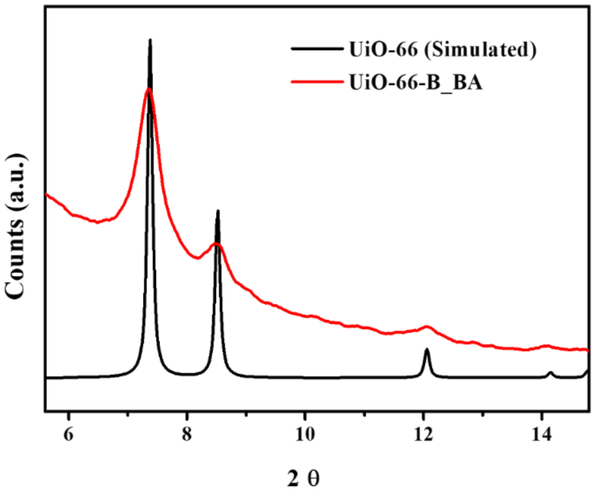

Figure S14. Comparison of powder X-ray diffraction pattern of UiO-66-B_BA, with the simulated pattern of UiO-66.

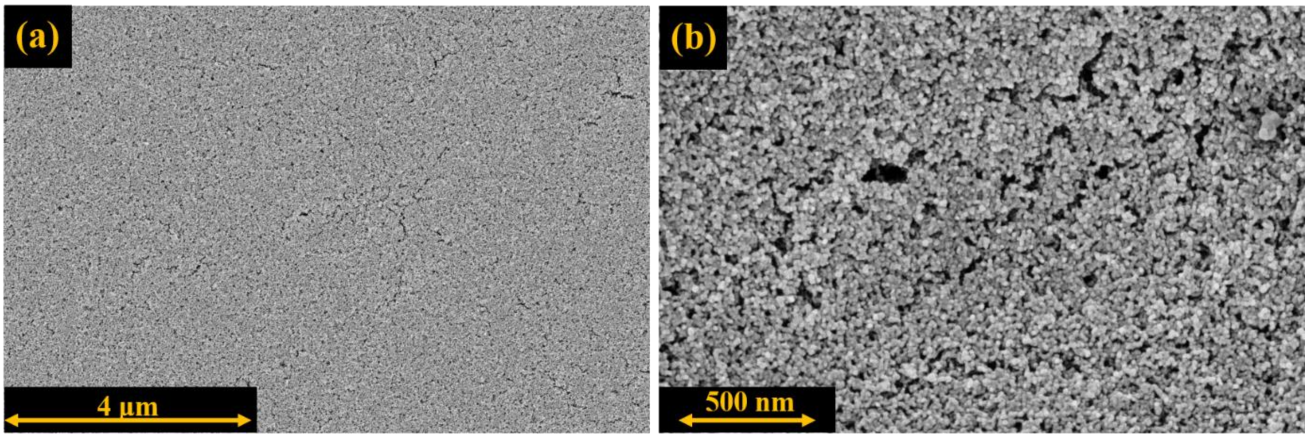

Figure S15. SEM images (top view) of UiO-66-B_BA at different magnifications showing the homogeneous nature of the UiO-66-B_BA thin film grown on Ag foil.

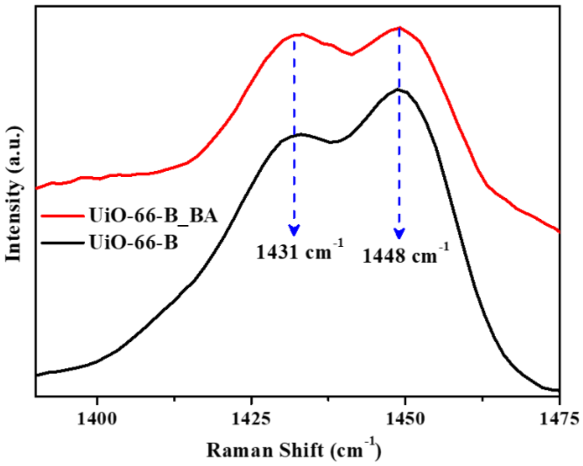

Figure S16. Raman spectra of the UiO-66-B (black) and UiO-66-B_BA (red). The two peaks at 1431 cm^{-1} and 1448 cm^{-1} of UiO-66-B suffers attenuation and alteration in terms of relative intensity for UiO-66-B_BA. These peaks appear due to the in-phase carboxylate (OCO) symmetric stretching of the BDC linkers for UiO-66 and due to the presence of both BDC linkers and BA in case of UiO-66-B_BA.

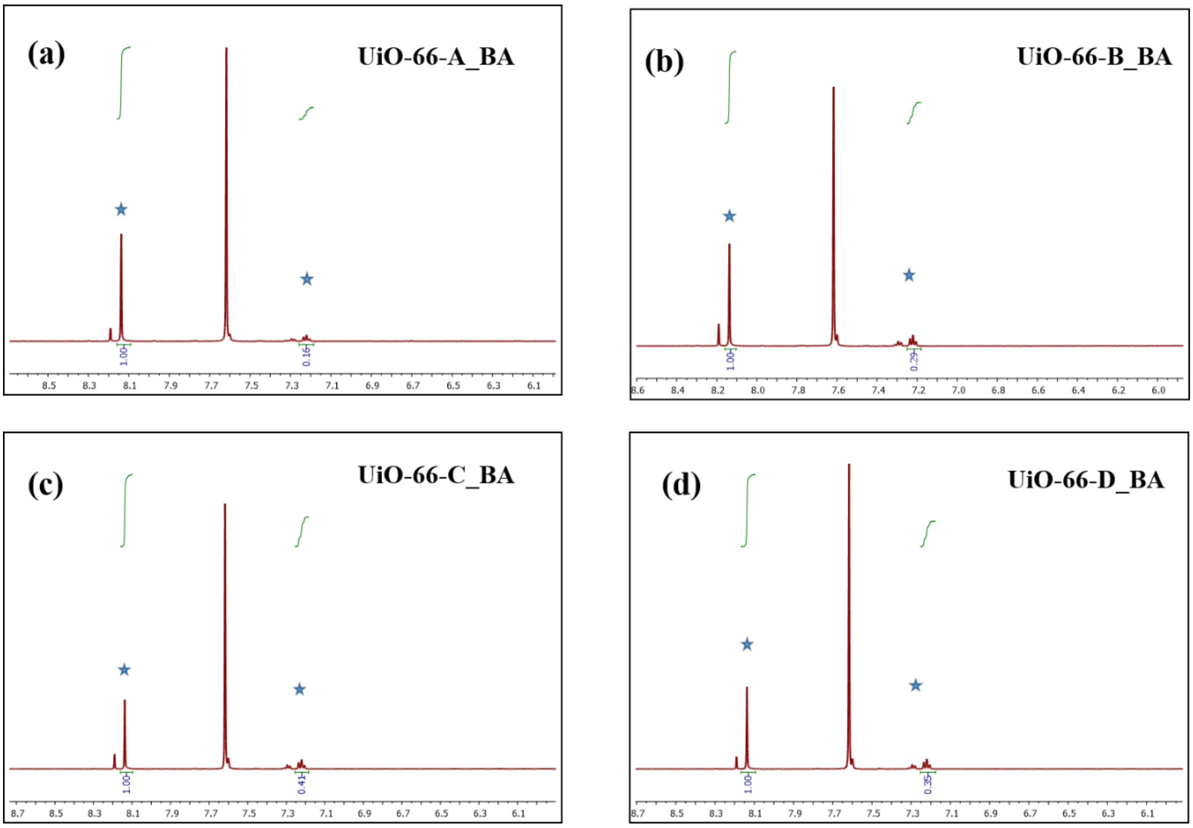

Figure S17. (a-d) ^{1}H-NMR spectra of UiO-66-(A-D)_BA digested in 2 M NaOH solution prepared in D_2O. Trimesic acid of known concentration was used as internal standard. The two peaks marked with * at ≈ 8.15 ppm and ≈ 7.2 ppm are attributed to the aromatic protons of the trime sic acid and the benzoic acid (BA) respectively.

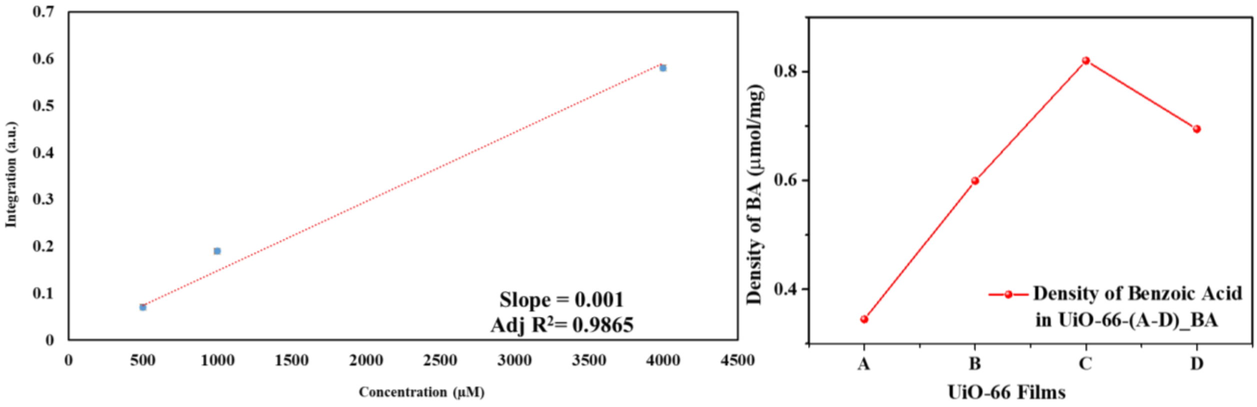

Figure S18. (Left) Calibration plot prepared by comparing the ^{1}H-NMR spectra of known concentration solutions of benzoic acid in 2 M NaOH prepared in D_2O. Trimesic acid of known concentration was used as internal standard. **(Right)** Loading of BA for UiO-66-(A-D)_BA as determined from the ^{1}H-NMR spectra of the UiO-66-(A-D)_BA (Figure S13) and calibration plot (Figure S14) of known amounts of benzoic acid (Figure).

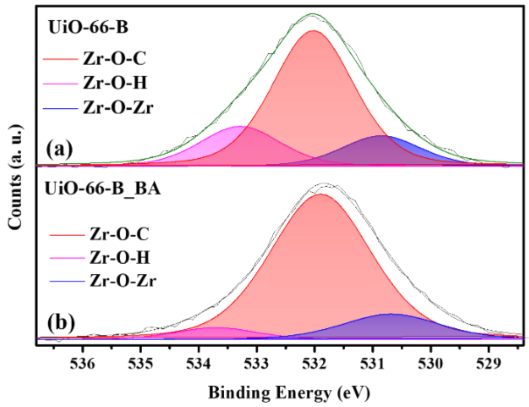

Figure S19. O1s X-ray photoelectron spectra of (a) UiO-66-B and (b) UiO-66-B_BA.

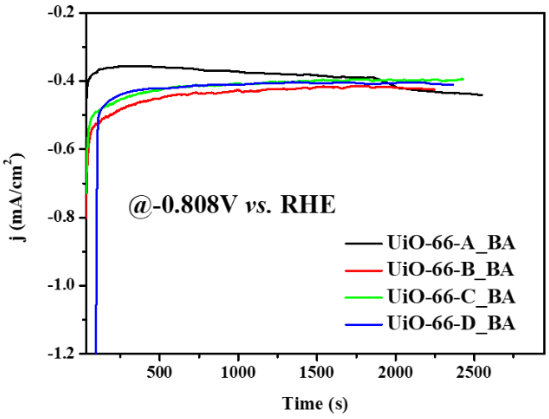

Figure S20. j (current density) *vs.* t (time) plot corresponding to the chronoamperometric measurements at -0.8 V (*vs.* RHE) for UiO-66-A_BA, UiO-66-B_BA, UiO-66-C_BA and UiO-66-D_BA.

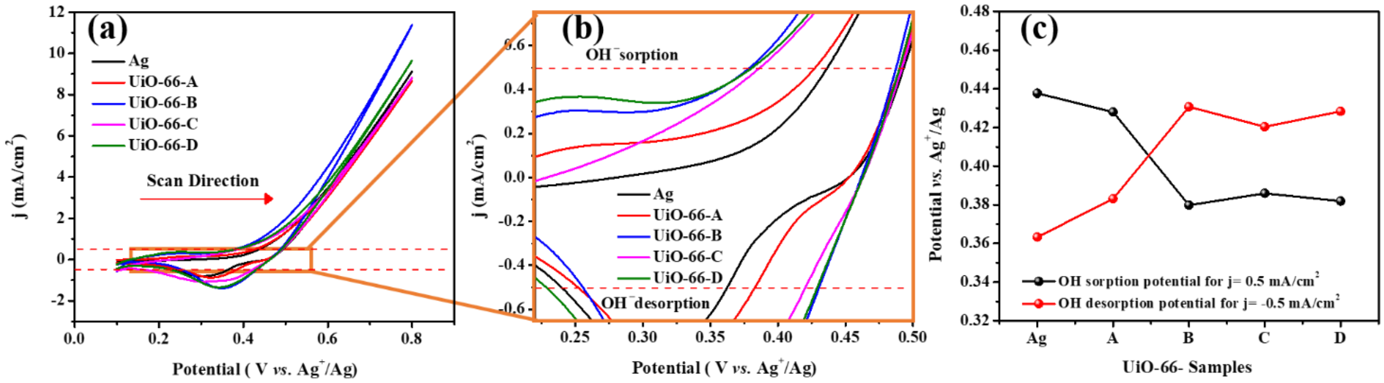

Figure S21. (a) Cyclic voltammograms of OH- reversible binding, recorded between 0.1 V and 0.8 V (*vs.* RHE) and (b) Zoomed-in portion of the cyclic voltammograms with cathodic and anodic current density marked at 0.5 mA/cm^2 for bare Ag, UiO-66-(A-D). Cyclic voltammograms were recorded using 0.1 M $NaClO_4$ of pH 9 (pH adjusted by 0.1 M NaOH), in one compartment cell with a scan rate of 50 mV/s. (c) shows the variation of the OH^- sorption and desorption potential for bare Ag and UiO-66-(A-D) at an anodic and cathodic current density of 0.5 mA/cm^2.

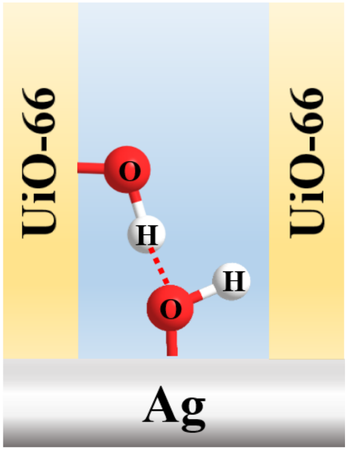

Figure S22. Illustration of the stabilization of Ag-bound *OH⁻ by secondary-sphere interaction with the dangling OH from the Zr_6-oxo metal nodes of UiO-66 thin film.

Effect of the UiO-66 thin film on the reversible binding of OH− on Ag surface.

Ag can reversibly bind OH− onto its surface. The activation energy for the absorption and desorption of Ag varies with the variation of Ag surface properties (Figure S17 and Figure S18). Now, the UiO-66-containing Ag surface is decorated with dangling OH from the Zr_6-oxo nodes adjacent to the metal cluster defect sites. These dangling groups can potentially stabilize the *OH^- onto the Ag surface, further lowering the required activation energy for OH^- sorption. For the same reason, the desorption may become more difficult in the UiO-66-containing Ag electrodes. This assumption matched well with the experimental results of the required potential for the sorption and desorption of OH^- on bare Ag and Ag electrodes modified with an UiO-66 thin film (Figure S17). The sorption and desorption potentials are presented in a tabular form (Table S3).

Table S4. 'OH^-' sorption and desorption potentials of bare Ag and UiO-66-(A-D) thin film coated Ag samples.

**Sample**	**Sorption Potential (V *vs.* Ag^+/Ag)**	**Desorption Potential (V *vs.* Ag^+/Ag)**
**Bare Ag**	0.436 V	0.363 V
**UiO-66-A**	0.427 V	0.383 V
**UiO-66-B**	0.379 V	0.430 V
**UiO-66-C**	0.385 V	0.420 V
**UiO-66-D**	0.381 V	0.428 V

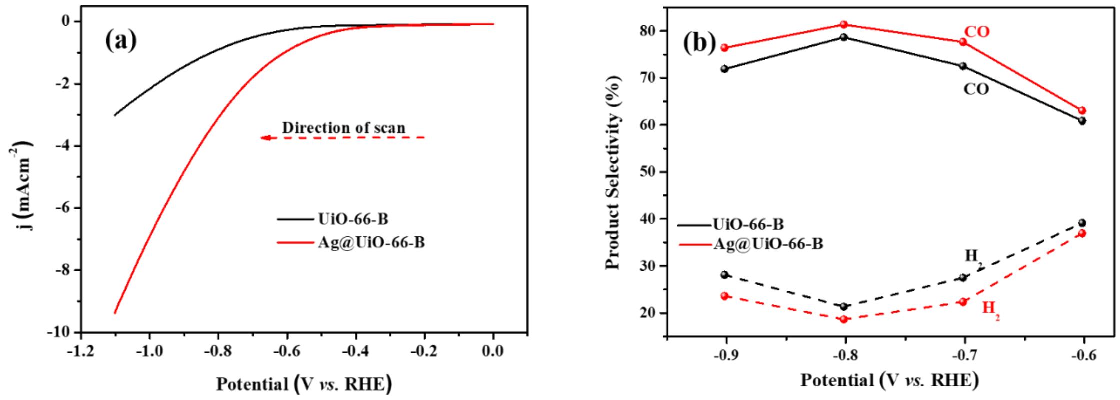

Figure S23. (a) Linear sweep voltammograms (LSVs) of UiO-66-B, and Ag@UiO-66-B. All measurements were done in CO_2-saturated 0.1 M $NaHCO_3$. Scan rate: 100 mV/s. (b) Variation in product selectivity of UiO-66-B and Ag@UiO-66-B towards CO and H_2 production at different applied potentials during electrocatalytic CO_2 reduction.

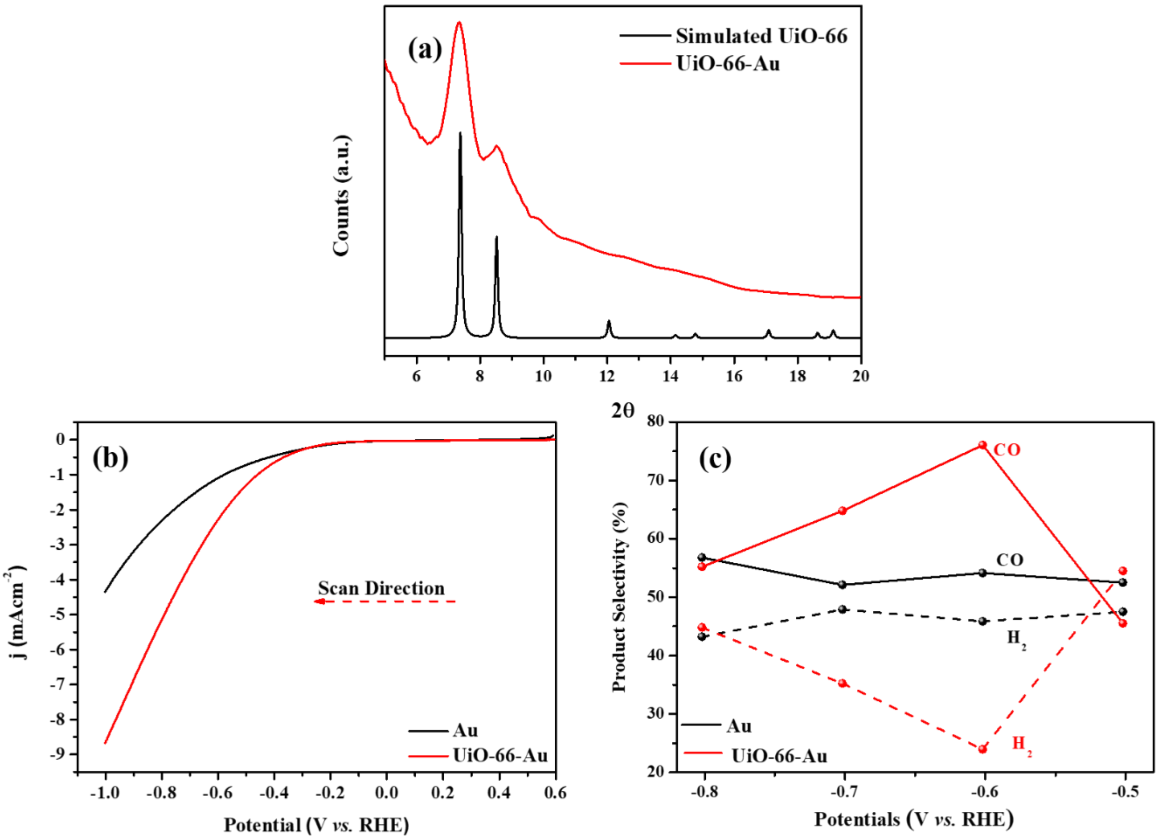

Figure S24. (a) PXRD patterns of UiO-66-Au compared with simulated PXRD pattern of UiO-66. (b) Linear sweep voltammograms (LSVs) of bare Au and UiO-66-Au. Measurements were done in CO_2-saturated 0.1 M $NaHCO_3$. Scan rate: 100 mV/s. (c) Variation in product selectivity of bare Au and UiO-66-Au towards CO and H_2 production at different applied potentials during electrocatalytic CO_2 reduction.

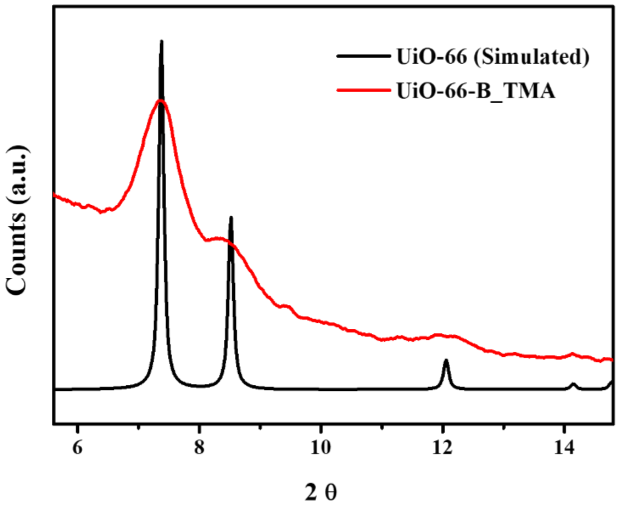

Figure S25. PXRD pattern of UiO-66-B_TMA compared with the simulated pattern of UiO-66.

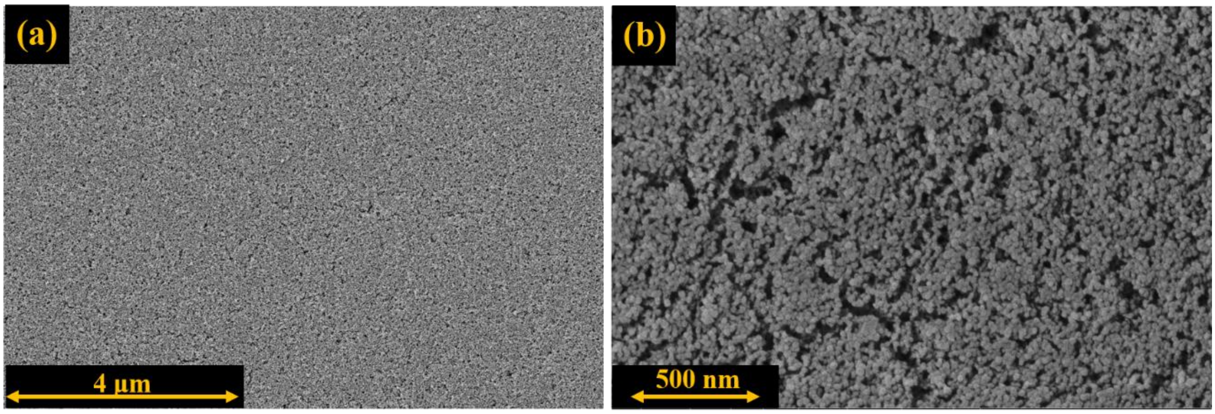

Figure S26. (a) and (b), SEM images (top view) of UiO-66-B_TMA thin film grown on Ag foil.

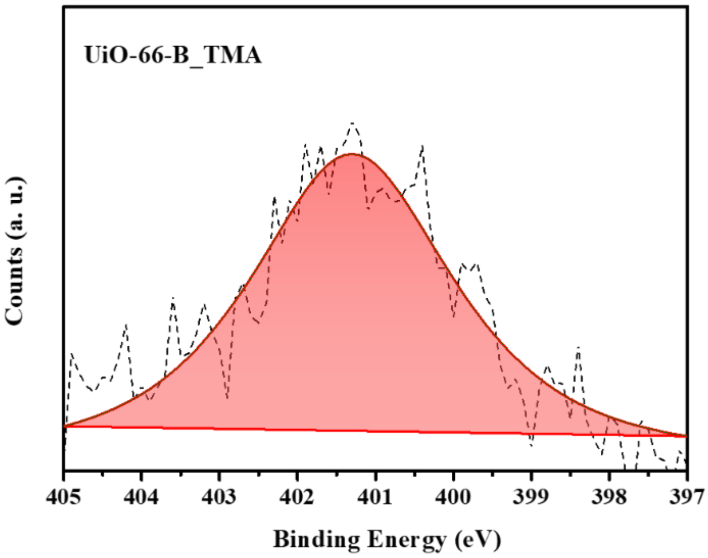

Figure S27. N1s X-ray photoelectron spectrum of UiO-66-B_TMA.

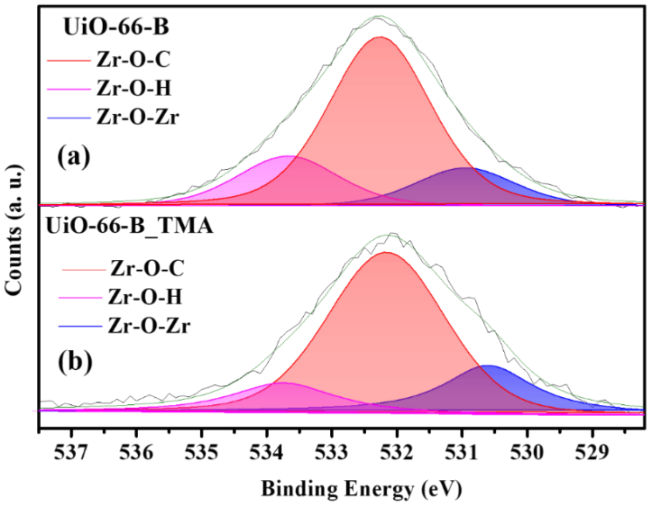

Figure S28. O1s X-ray photoelectron spectra of (a) UiO-66-B and (b) UiO-66-B_TMA.

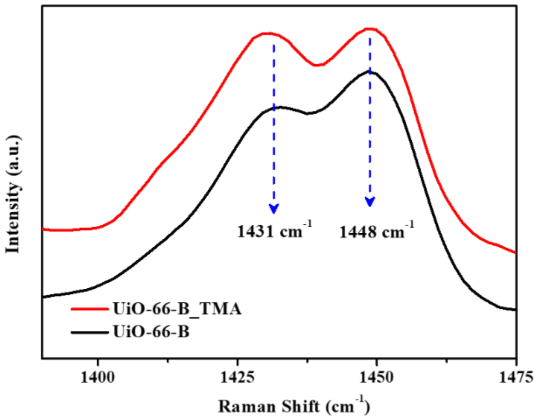

Figure S29. Raman spectra of the UiO-66-B (black) and UiO-66-B_TMA (red). The two peaks at 1431 cm^{-1} and 1448 cm^{-1} of UiO-66-B suffers attenuation and alteration in terms of relative intensity for UiO-66-B_TMA. These peaks appear due to the in-phase carboxylate (OCO) symmetric stretching of the BDC linkers for UiO-66 and due to the presence of both BDC linkers and TMA in case of UiO-66-B_TMA.

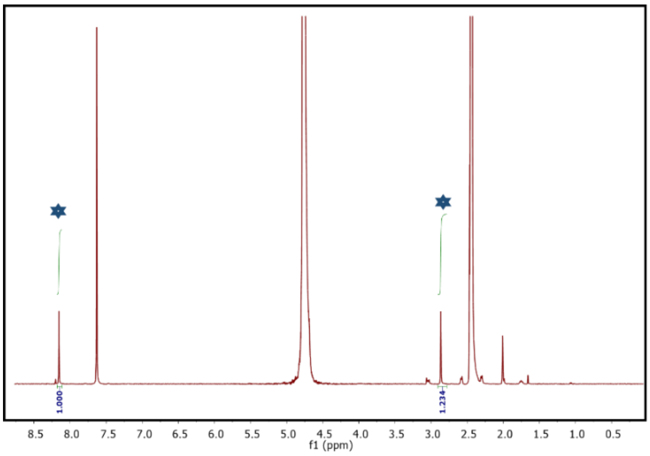

Figure S30. ^{1}H-NMR spectrum of known amount of UiO-66-B_TMA digested in 2 M NaOH solution prepared in D_2O. Trimesic acid of known concentration was used as internal standard. The two peaks marked with * at ≈ 8.15 ppm and ≈ 2.8 ppm are attributed to the aromatic protons of the trimesic acid and the methyl ($-CH_3$) protons of the TMA ligand, respectively.

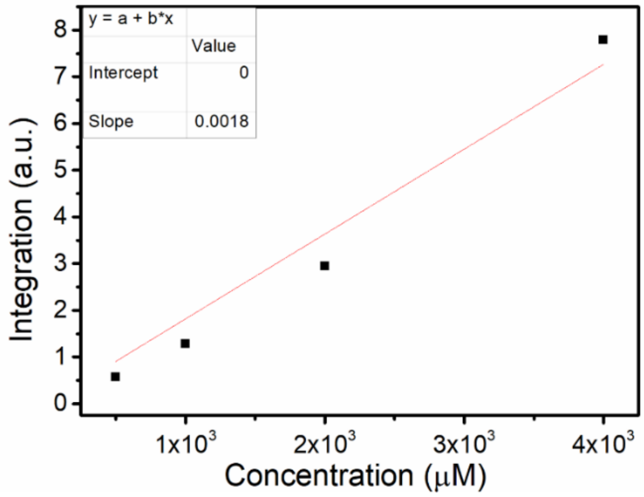

Figure S31. Calibration plot prepared by comparing the relative intensity of methyl (-CH_3) protons in the ^{1}H-NMR spectra of known concentration solutions of TMA in D_2O. Trimesic acid of known concentration was used as internal standard.

Determination of the loading level of TMA ligand from NMR combined with ICP-OES analysis.

For ICP-OES analysis, known amount of UiO-66-B_TMA were digested in 5 ml of conc. HNO_3 at 150 ℃ for 12 hours. 1 ml of the acid mixture was diluted to 10 ml by adding water and analyzed by ICP-OES.

According to ICP-OES analysis, the concentration of Zr^{4+} in the solution was 8.708×10^{-6} g/ml. Thus, total amount of Zr in 2 mg UiO-66-B_TMA is $(8.708 \times 10^{-6} \times 50)$ g. 2 mg of UiO-66-B_TMA contains $[(8.708 \times 10^{-6} \times 50)/547.32]$ mole ≈ 0.8 μmol. of Zr_6 units. To determine the loading level of the TMA ligand, ^{1}H-NMR was recorded for a solution prepared by dissolving 2 mg of UiO-66-B_TMA in 500 μl of 2 M of NaOH, prepared in D_2O (Figure S24). A calibration plot was also derived using known concentrations of TMA solutions prepared in D_2O (Figure S25). Trimesic acid was used as the internal standard in all the cases.

According to the calibration plot, the concentration of TMA ligand of the MOF solution was 685.5 μM. Thus, the 2 mg of UiO-66-B_TMA has (685.5 μmol/l × 500 μl) = 0.343 μmol of TMA ligand.

So, the loading level of TMA per Zr_6 unit in case of UiO-66-B_TMA is (0.343/0.8) = 0.428 ≈ 0.4 (approx.). In other words, 2 TMA ligand is present for every 5 Zr_6 metal nodes in UiO-66-B_TMA.

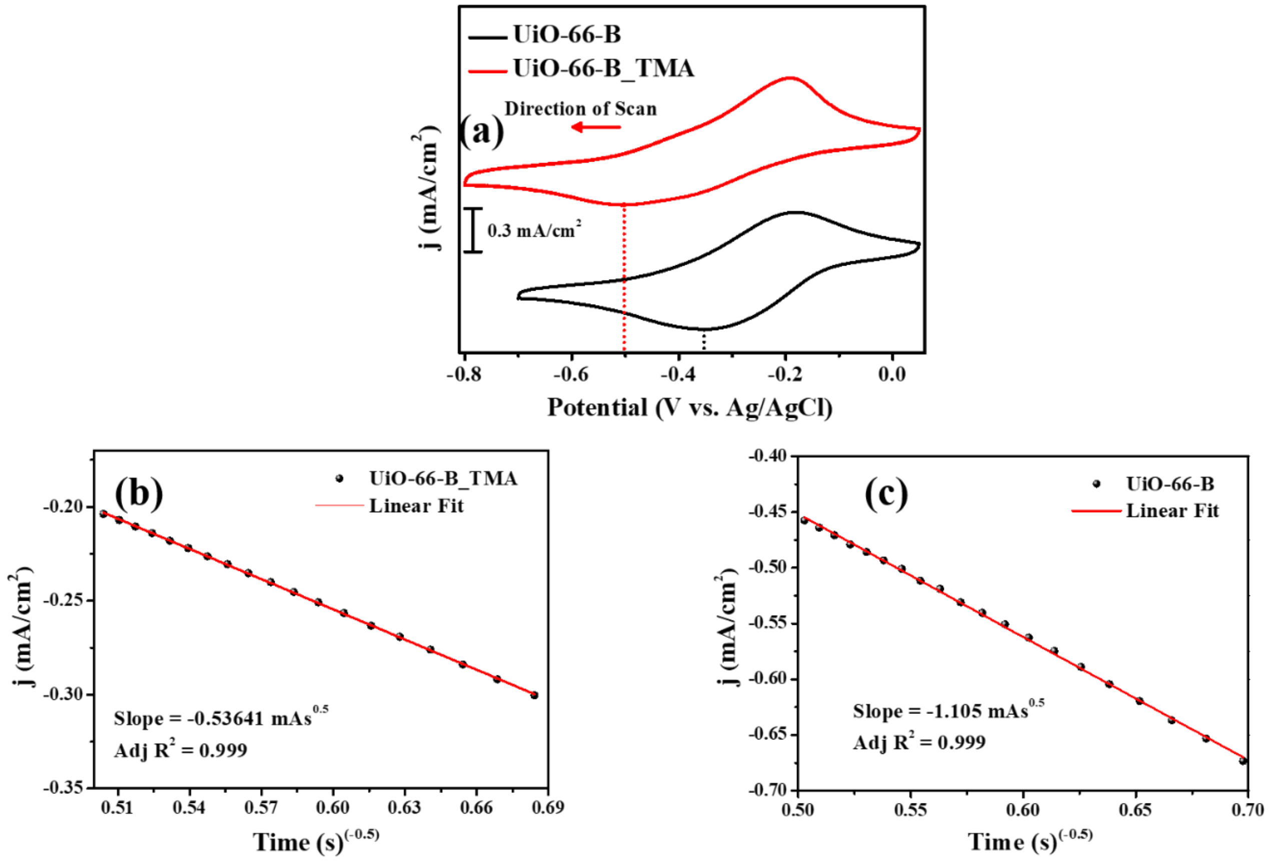

Figure S32. (a) Cyclic voltammograms of UiO-66-B and UiO-66-B_TMA recorded in an Ar-saturated aqueous solution consisting of 10 mM of $[Ru(NH_3)_6]Cl_3$ and 0.1 M of $NaClO_4$. Scan rate: -100 mV/s. (b) and (c) show the Cottrell plot (current density vs. inverse square root of time) for UiO-66-B and UiO-66_TMA, respectively.

Determination of diffusion coefficient.

The cyclic voltammograms of UiO-66-B and UiO-66-B_TMA show that the cathodic wave corresponding to the $[Ru^{III}(NH_3)_6]^{3+}$ → $[Ru^{II}(NH_3)_6]^{2+}$ conversion is shifted towards further cathodic potentials for UiO-66-B_TMA than for UiO-66-B (Figure S26.a). This could be because of the presence of the positively charged TMA ligand across the UiO-66-B-TMA thin film, which can result into attenuated diffusion of the $[Ru^{III}(NH_3)_6]^{3+}$ cationic probe to the Ag electrode surface, because of electrostatic interactions.

From the potential-step chronoamperometric measurements, the diffusion coefficient (D) for $[Ru^{III}(NH_3)_6]^{3+}$ was determined for both UiO-66-B and UiO-66-B_TMA. The resulting current transients obey the Cottrell relation (eqn. 1) and the diffusion coefficient can be determined from the slope of the plot between current density (j) and (time (t))$^{-0.5}$ (Figure S26)

Equation 1.

$$\mathbf{i(t)} = \frac{nFAC\sqrt{D}}{\sqrt{\pi t}}$$

Where, i(t) = current; n = number of electrons transferred = 1 in this case; F = 1 Faraday = 96500 C (approx.); A = geometrical area of the working electrode = 1 cm^2 in this case; C = concentration of the redox active $[Ru^{III}(NH_3)_6]^{3+}$ (in mol/cm^3) = 1×10^{-4} mol/cm^3; D = diffusion coefficient (in cm^2/s).

At short measurement time scales, the diffusion obeys semi-infinite conditions.

Thus, i(t) *vs.* $t^{-1/2}$ behaves linearly, and the value of D can be extracted from the curve's slope (Figure S26. b and c). Thus, the Diffusion constant for $[Ru^{III}(NH_3)_6]^{3+}$ in case of UiO-66-B and UiO-66-B_TMA was determined. $D_{UiO\text{-}66\text{-}B}$ is about 4 times larger than $D_{UiO\text{-}66\text{-}B\_TMA}$ (Table S4).

Table S5. Diffusion coefficients (D) of UiO-66-B and UiO-66-B_TMA.

Sample	Slope ($mA/s^{0.5}$)	Diffusion coefficient (D) (cm^2/s)
**UiO-66-B**	-1.105	41×10^{-9}
**UiO-66-B_TMA**	-0.5364	9.6×10^{-9}

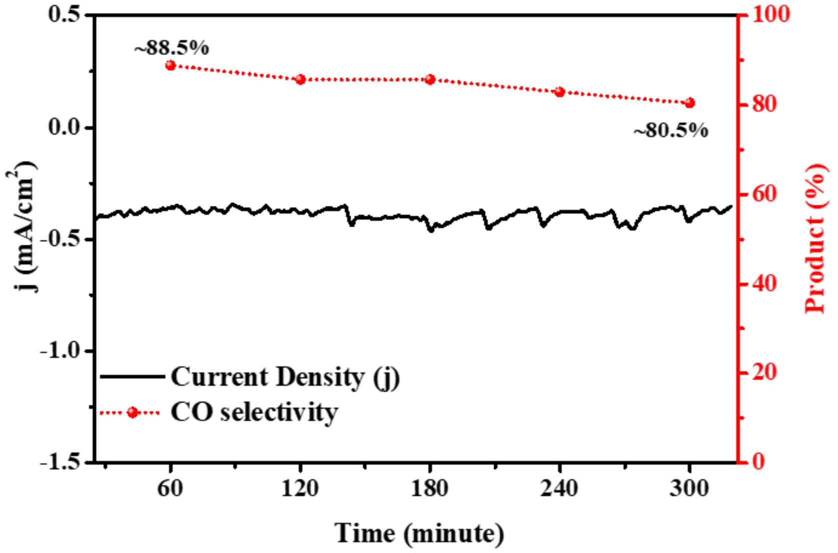

Figure S33. Variation in catalytic current density and CO selectivity during a 5-hour chronoamperometric measurement of UiO-66-B_TMA in CO_2-saturated 0.1 M $NaHCO_3$ solution. To maintain the CO_2 saturation, the solution was purged every hour with CO_2 for 30 minutes and sealed before continuing the measurement further. The measurement was performed in a two-compartment cell.

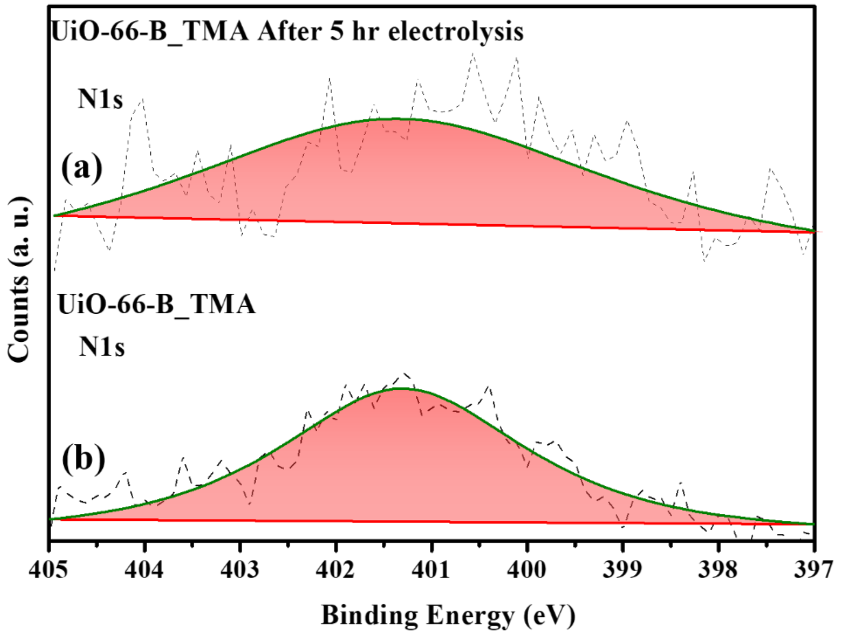

Figure S34. N1s X-ray photoelectron spectra of (a) UiO-66-B_TMA after 5 hours of electrolysis at -0.8 V (*vs.* RHE) in CO_2-saturated 0.1 M $NaHCO_3$ and (b) UiO-66-B_TMA.

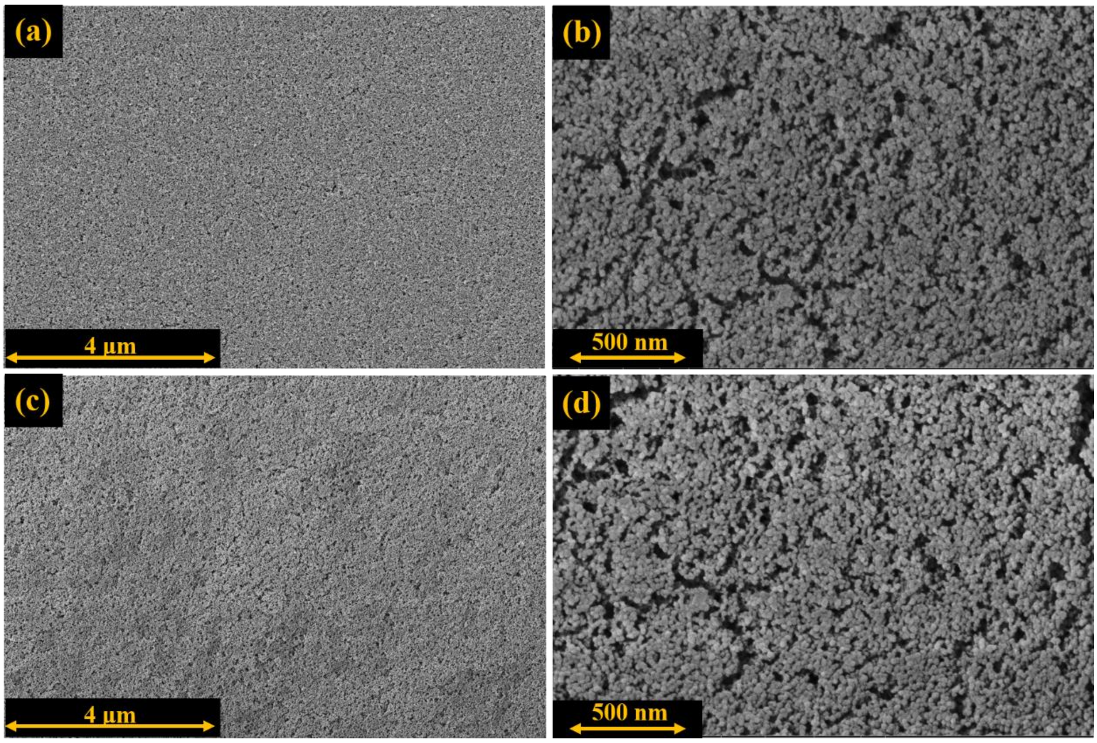

Figure S35. {(a) and (b)}, SEM images (top view) of UiO-66-B_TMA thin film grown on Ag foil; {(c) and (d)} SEM images (top view) of UiO-66-B_TMA after 5 hours of electrolysis at -0.8 V (*vs.* RHE) in CO_2-saturated 0.1 M $NaHCO_3$.

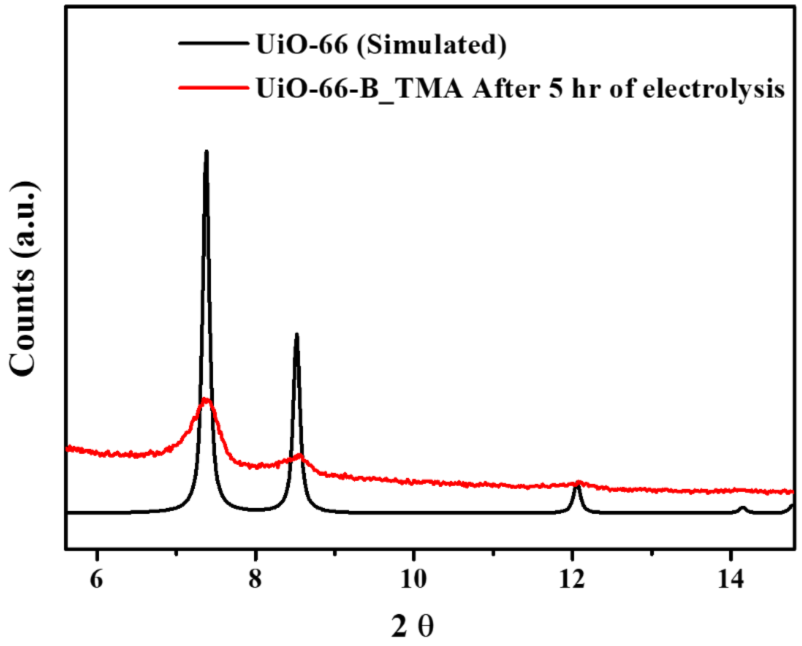

Figure S36. Comparison of the PXRD pattern of UiO-66-B_TMA after 5 hours of electrolysis at -0.8 V (*vs.* RHE) in CO_2-saturated 0.1 M $NaHCO_3$ with the simulated pattern of UiO-66 to understand the structural integrity of the MOF thin film after electrolysis.

Author Contributions

Idan Hod supervised this project. Subhabrata Mukhopadhyay carried out the project. Subhabrata Mukhopadhyay conducted experiments and analyzed data. Ran Shimoni performed the XRD measurements, N_2 sorption measurements and helped with gas-chromatography. Itamar Liberman performed the ICP-OES measurements and SEM measurements. Raya Ifreamov performed the Raman measurements. Ran Shimoni and Illya Rozenberg performed the NMR tests and analyzed the data. Idan Hod and Subhabrata Mukhopadhyay wrote the paper.
